# A novel design to screen chlorogenic acid-producing microbial strains from the environment

**DOI:** 10.1038/s41598-018-32968-0

**Published:** 2018-10-03

**Authors:** Xiao Wang, Lifang Qin, Jia Zhou, Youzhi Li, Xianwei Fan

**Affiliations:** State Key Laboratory for Conservation and Utilization of Subtropical Agro-bioresources, College of Life Science and Technology, Guangxi University, Nanning, 530004 China

## Abstract

The present study aimed to develop a plate-screening method, based on the specific color development of complexes formed between chlorogenic acid, a valuable plant-derived compound, and aluminum (III), to detect chlorogenic acid-producing microbial strains. Modified media with 0.75 mM aluminum chloride were developed to identify CGA-producing bacteria (based on beef extract agar medium) or fungi (based on the potato dextrose agar medium). Compared with conventional screening, the modified media let to 3.3 times more CGA producers from plants, at 90.9% selective accuracy. Novel chlorogenic acid-biosynthesizing strains included *Brevibacillus borstelensis* B14, *Bacillus amyloliquefaciens* B17, *Bacillus badius* B19, *Sphingomonas yabuuchiae* N21, *Enterobacter tabaci* N22, and *Lodderomyces elongisporus* S216 and P212. Strain S216 produced the highest chlorogenic acid yield (23.39 mg L^−1^). This study provides a highly efficient and low-cost tool for quick detection and subsequent identification of several newly isolated strains with chlorogenic acid-producing potential.

## Introduction

Chlorogenic acid (CGA) is the major soluble phenolic compound formed via the shikimic acid pathway in almost all plants, and its structure was first established as 3-O-caffeoylquinic acid by Fischer and Dangschat^1^. The biosynthesis of CGA involves three different possible pathways depending on the plant species: The intermediate production of an ester of quinic acid and caffeic acid, a cinnamyl-quinic ester formed during CGA production from phenylalanine in potato^2,3^, or via the activated acid, caffeoyl glucoside^4^. CGA is also an important active ingredient found in a wide variety of plants, fruit, and vegetables and is considered beneficial for human health^5^. In particular, CGA can accumulate at high levels in traditional Chinese medicinal materials, such as *honeysuckle* and *Eucommia ulmoides*^6^. Studies have shown that CGA possesses more powerful antioxidant activities in comparison to many flavonoids and has a broad range of applications in preventing cardiovascular and age-related diseases, protecting against ischemia-reperfusion injury^3,7,8^, which was attributed to scavenge free radicals^9^. CGA has been also recognized to inhibit carcinogenesis in the colon and liver^10–12^, improve lipid metabolism in mice^13^, and exert anti-bacterial, anti-inflammatory, and anti-diabetic activities^14,15^. Furthermore, the high CGA content in plants can enhance the resistance to pathogenic infection^16^.

The current demand for natural CGA has a potential growing trend in the field of medicine, chemicals, food preservation, and cosmetic applications^17^. The production of CGA derives mainly from extracts from medicinal plants, however, the extract of plants usually limits the CGA production due to the low content in plants and longer growth cycle. Application of chemical modification and structural transformation will bring about the serious pollution to environment. Recently, certain microbes can biosynthesize CGA by the esterification of quinic acid and caffeic acid in the secondary metabolic pathway, including bacteria^18^ and endophytic fungi^19^. Therefore, efforts have been made to isolate microbes with the ability to produce CGA. However, CGA is usually identified using high-performance liquid chromatography (HPLC), which is not suitable for screening large numbers of microbes from the environment because the method is laborious, expensive, and time consuming. Therefore, a simple method to rapidly detect CGA-producing fungi and bacteria is necessary.

In the pH range of 6.5–7.5, Al^3+^ can coordinate via the phenolate groups or the carboxyl group of CGA^20^, as well as the catecholate moiety, to form a relative stable CGA-Al^3+^ complex^20,21^. The chromogenic-complexing reaction of Al^3+^ and CGA has been applied to determine the level of CGA in plant leaves under alkaline conditions^21^. Therefore, the aim of this study was to evaluate the ability and efficacy of a newly designed Al^3+^-containing media to isolate CGA-biosynthesizing microbes.

## Results

### The characteristics of chlorogenic acid-Al^3+^ complexes in a pH range 6.0–7.5 and the solidification of modified potato dextrose agar medium

**Figure 1:**
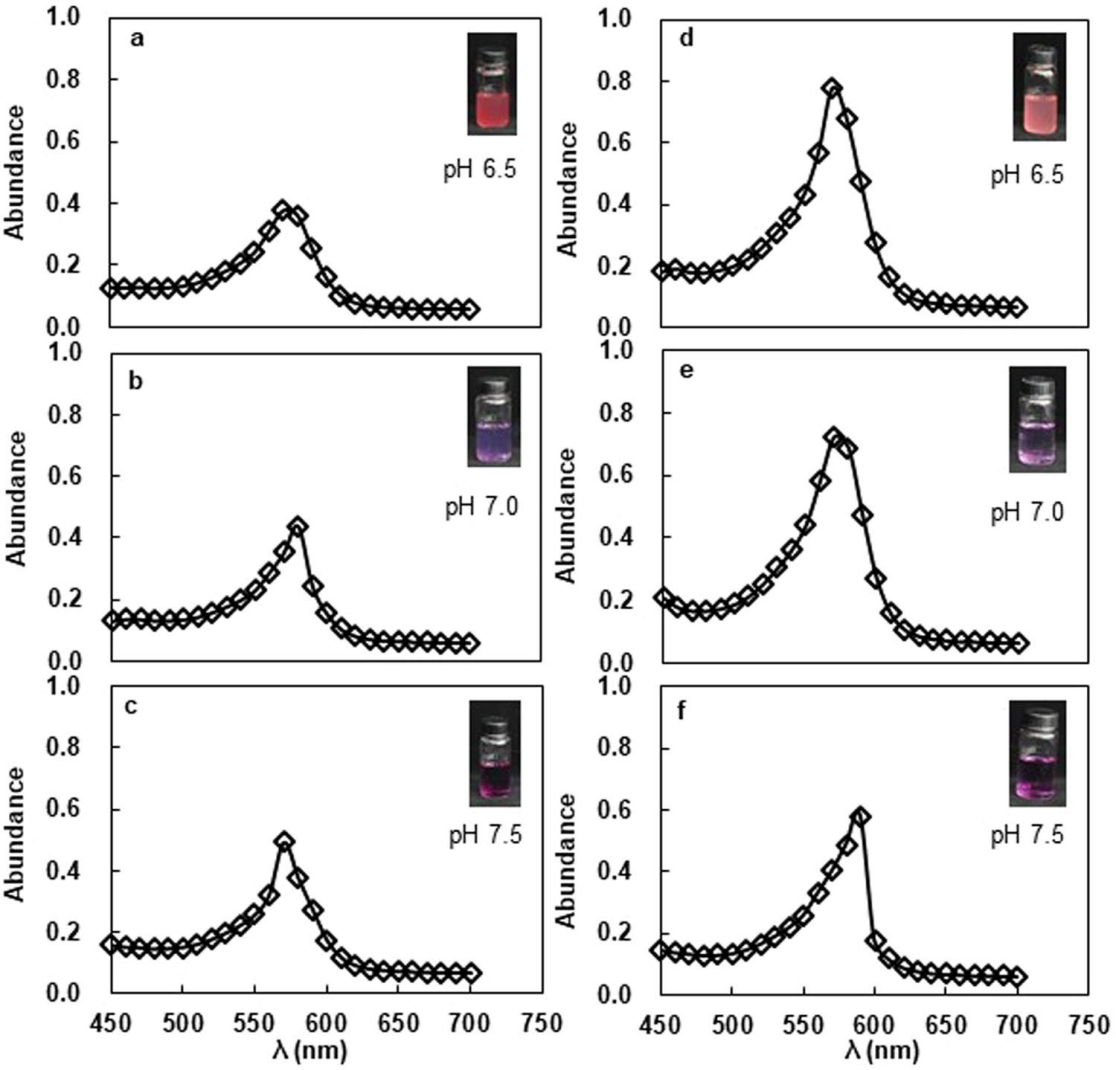
The absorption spectra and color development of the complex formed between chlorogenic acid and aluminum (III) in the pH range of 6.5–7.5 in water solution (**a**–**c**) and in beef extract agar (BEA) liquid medium (**d**–**f**). [Al] = 0.9 mM, [chlorogenic acid] = 1.8 mM.

**Figure 2:**
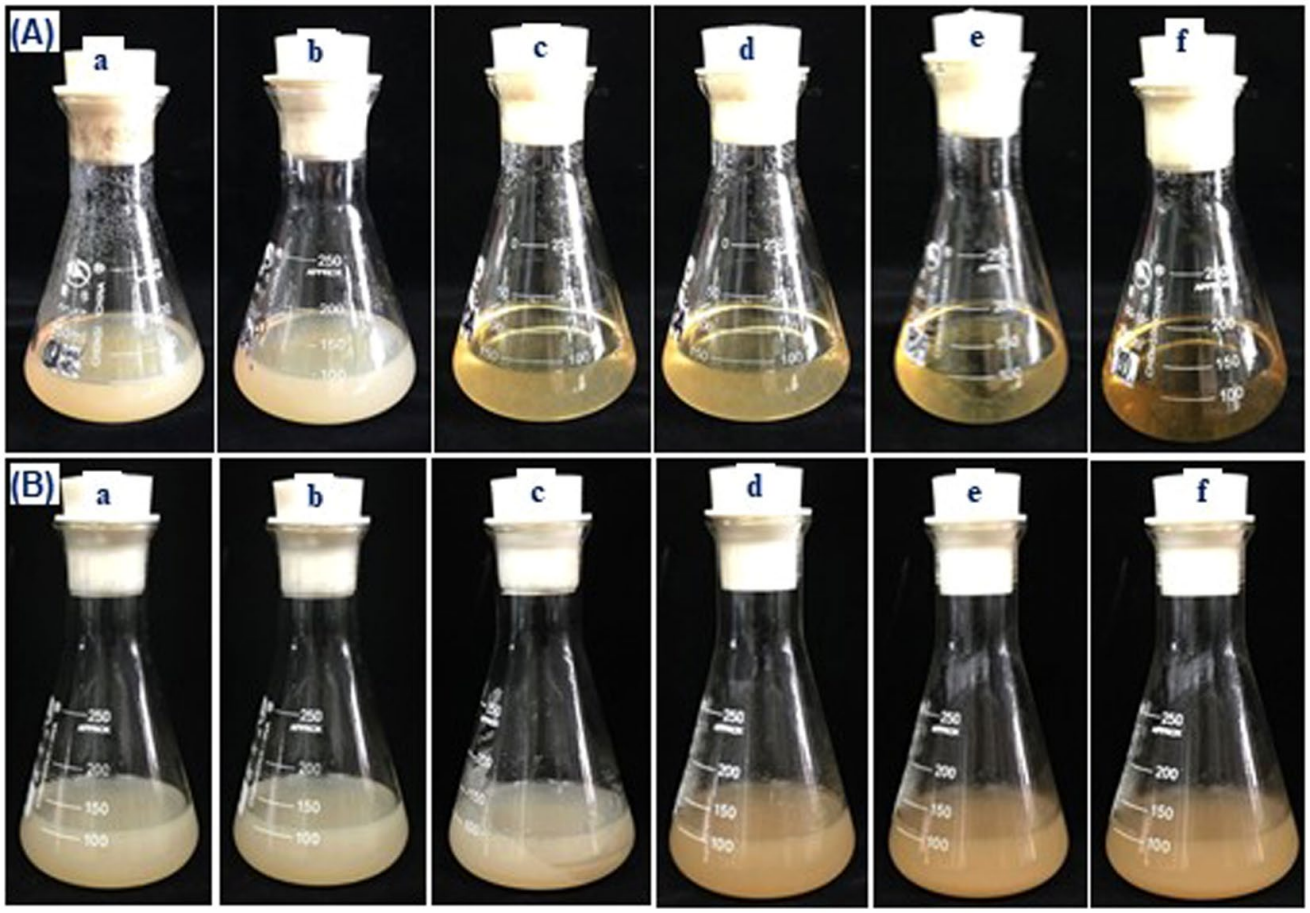
The solidity of potato dextrose agar (PDA) (**A**) and BEA (**B**) culture medium supplement with different concentrations of AlCl_3_ ranging from 0.375 to 15 mM. (**a**) 0.375 mM; (**b**) 0.75 mM; (**c**) 1.5 mM; (**d**) 3.75 mM; (**e**) 7.5 mM; (**f**) 15 mM.

A single absorption peak was observed at 570 nm after formation of the CGA-Al^3+^ complex at pH values of 6.5–7.5 (Fig. 1). The color developed gradually from pink to dark purple as pH increased in the aqueous solution of CGA and aluminum (Fig. 1a–c). The absorption peak was similar when the reaction solution was changed from water to liquid beef extract agar (BEA) medium (Fig. 1d–f), and the color development of the complex formation faded slightly in the liquid BEA medium. Similar results were obtained in the liquid PDA medium (data not shown). Other structural similar compounds (caffeoylshikimic acid, shikimic acid, quinine acid, caffeic acid, trans-cinnamic acid and p-coumaric acid) were also used to check the chelating ability with AlCl_3_ at pH 7.0, but no absorbance and color development were observed in the mixture solution, except for yellow changes in caffeoylshikimic acid solution (Fig. S1). The solidification of PDA culture medium was determined following supplementation with AlCl_3_ at pH 7.0. Modified medium could be solidified with the addition of 0.375 and 0.75 mM AlCl_3_ (Fig. 2Aa,b), but remained in a semi-solid state when 1.5–3.75 mM AlCl_3_ was supplemented (Fig. 2Ac,d); however, the medium did not solidify at 7.5–15 mM AlCl_3_ (Table 1 and Fig. 2Ae,f). The effects of AlCl_3_ on the solidification of BEA culture medium were similar with those observed for PDA culture medium (Fig. 2B).

**Table 1:** Effect of the supplemented AlCl_3_ concentration on the solidification of PDA culture medium at pH 7.0.

AlCl_3_ concentration (mM)	Medium reaction	Color development
0.375	solidified	faint yellow (slow)
0.750	solidified	violet-red (fast)
1.500	semi-solidified	—
3.750	semi-solidified	—
7.500	non-solidified	—
15.000	non-solidified	—

Note: —Indicates no color response.

**Figure 3:**
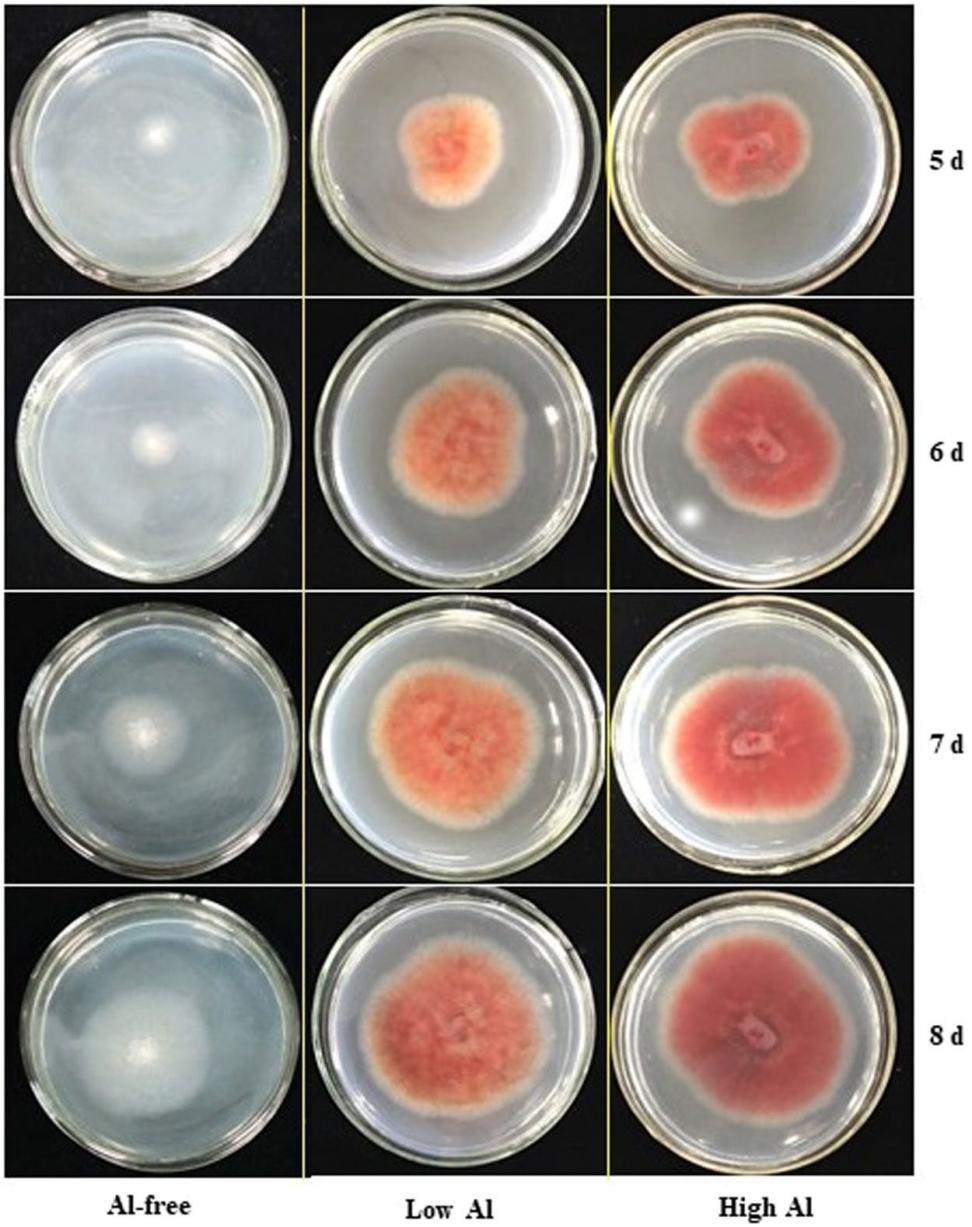
The color development and size of the stained circle for strains capable of producing chlorogenic acid on potato dextrose agar (PDA, Al-free) and modified PDA culture medium with low (0.375 mM) and high (0.75 mM) Al after culture.

BEA and PDA media (basal media) were each supplemented with 0.375–0.75 mM AlCl_3_ (modified media) to assess the color development after inoculation with CGA-producing strains. Compared with Al-free medium, the rate of color formation and change in coloration were significantly different between media containing high and low levels of aluminum ions during the screening process. As an example, the color of the halo produced by strain S216 was faint yellow on PDA plates with the addition of 0.375 mM aluminum chloride, and the color darkened with extended culture time (Fig. 3). Modified medium with 0.75 mM aluminum chloride produced a violet-red halo that remained stable during the extended culture period (Table 1 and Fig. 3). The color diffusion diameter on the PDA culture plate with the higher concentration of Al^3+^ was larger than those obtained with the lower Al^3+^ concentration and Al-free medium (Fig. 3 and Table S1). Similar results were obtained using BEA media (data not shown).

### Screening efficiency of modified medium supplemented with 0.75 mM and basal medium

To establish the effectiveness of the modified medium, leaves from seven plant species, *Ipomoea batatas*, *Eucommia ulmoides* Oliver, *Lonicera japonica*, *Camellia*, *Lonicera hypoglauca*, *Mentha haplocalyx*, and *Laurocerasus phaeosticta*, were each cleaned with 70% ethanol for surface sterilization and then excised pieces from them were inoculated into basal media (BEA or PDA) and modified media containing aluminum to screen strains from many species (Table 2). All of the strains were then assessed for their ability to produce CGA using HPLC. Among the leaves from the seven plants, 106 strains (bacteria or fungi) were isolated from basal media, and only 11 strains (bacteria or fungi) were isolated from modified media based on the color halo of isolates (Table 2). However, only three bacteria among the 106 strains isolated from the basal media were found to produce CGA. Using the modified media, four bacteria and six fungi among the 11 strains were found to produce CGA, and the average selective accuracy was almost 90.9% on the modified medium (Table 2).

**Table 2:** Comparison of % CGA producers between basal medium and modified medium used to isolate endophytic strains able to produce chlorogenic acid.

Plants species	Basal Medium					Modified Medium						
	TN of bacteria^#^	TN of fungi*	CGA bacteria	CGA fungi	% CGA producers	No. of Bacteria^§^	No. of Fungi^§^	CGA bacteria	CGA fungi	TN of bacteria^#^	TN of fungi*	% CGA producers
*Ipomoea batatas*	24	8	1	0	3.1%	1	2	1	2	4	3	100%
*Eucommia ulmoides Oliver*	16	4	0	0	0%	0	3	0	2	3	4	66.7%
*Lonicera japonica*	6	7	1	0	7.7%	1	0	1	0	3	1	100%
*Camellia*	6	6	0	0	0%	0	1	0	1	1	1	100%
*Lonicera hypoglauca*	6	3	0	0	0%	0	1	0	1	1	1	100%
*Metha haplocalyx*	9	5	1	0	7.1%	2	0	2	0	4	0	100%
*Laurocerasus phaeosticta*	4	2	0	0	0%	0	0	0	0	1	0	100%

Note: TN = total number; pound key ^#^and asterisk ^*^mean colonies growing on BEA and PDA plates; ^§^indicates the number of colonies producing colored-halos. CGA-biosynthetic ability of all colonies growing on basal medium and modified medium were determined by TLC and HPLC method.

Two additional strains 261 and 262 were isolated from the rhizosphere soil of herbs in Guangxi province using color development on the modified medium, and were found to biosynthesize CGA, as assessed using HPLC. Following the analysis of their 16S rDNA sequences, they were separately confirmed as *Streptomyces* sp. and *Serratia* sp. (Supporting Information 1).

### Evaluation of selected strains with the ability to produce chlorogenic acid

The positive strains from the modified medium and the strains from the basal medium were further analyzed using TLC. The R_f_ of authentic CGA was about 0.75, and the extracts of 13 strains (all chromatographic data are not shown) had similar R_f_ values to that of authentic CGA (Fig. S2 and Table S2). Three positive strains (N23, B111, N224) were isolated from the basal medium, and the Rf of N23, B111 and N224 were respectively 0.74, 0.76 and 0.74; 10 other strains (P212, P292, P2102, B14, B17, B19, S216, N21, N22, D215) were isolated from modified medium (Table S2), and the Rf of P212, P292, P2102, B14, B17, B19, S216, N21, N22 and D215 were 0.74, 0.73, 0.75, 0.74, 0.74, 0.77, 0.75, 0.74, 0.75 and 0.75, respectively.

**Figure 4:**
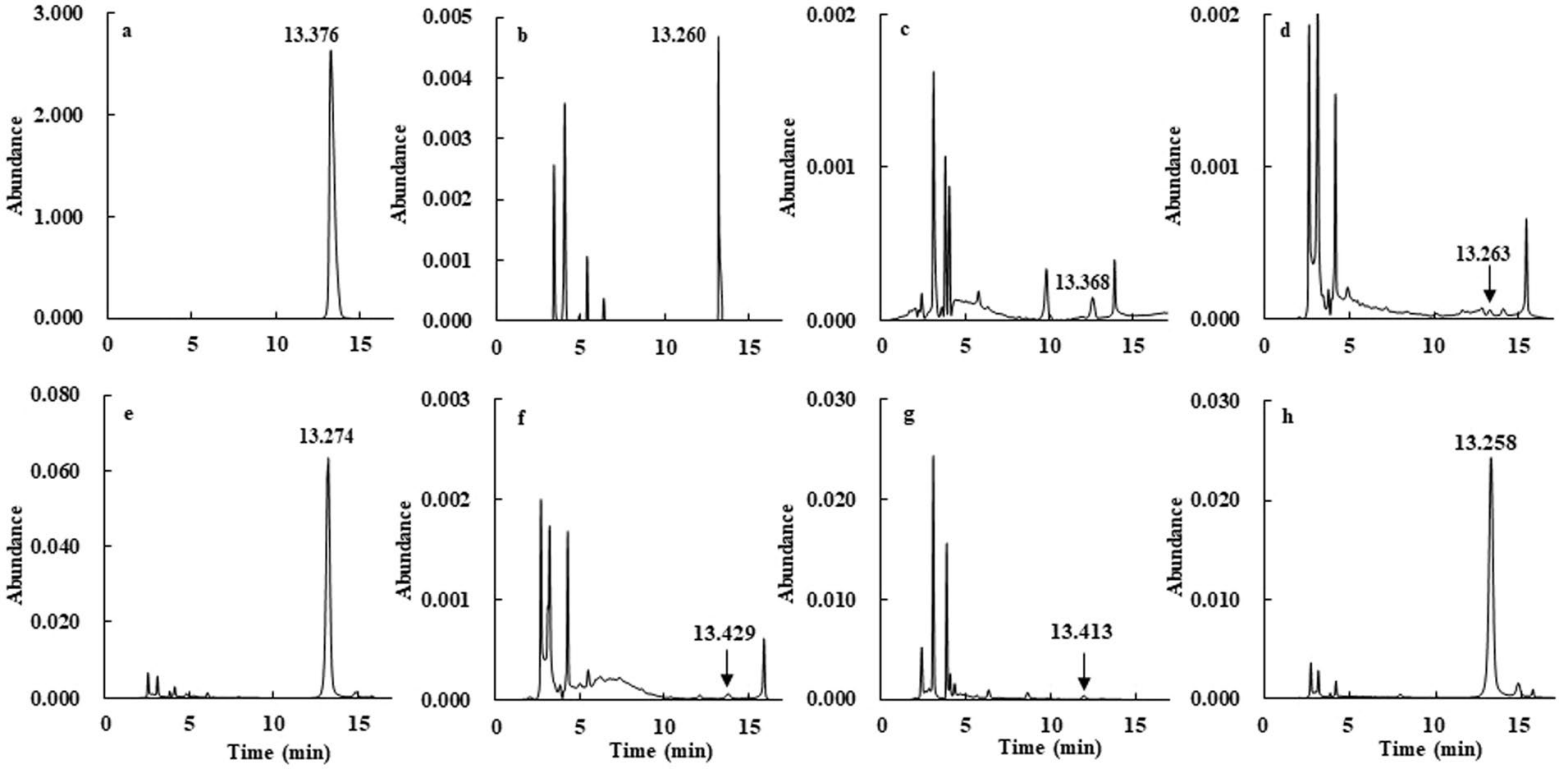
High performance liquid chromatography (HPLC) chromatography results of authentic chlorogenic acid (**a**) and the extracts from chlorogenic acid-positive strains (**b**–**h**). The mobile phase comprised 0.5% acetic acid and acetonitrile (92:8, v/v). The retention time of authentic chlorogenic acid was 13.376 min, and the retention times of the analytes from the extracts from chlorogenic acid-positive strains were 13.260 min for B14 (**b**) 13.369 min for B17 (**c**) 13.263 min for B19 (**d**) 13.274 min for N21 (**e**) 13.430 min for N22 (**f**) 13.413 min for P212 (**g**) and 13.258 min for S216 (**h**).

To ensure the accuracy of the TLC results, samples from the selected strains were further analyzed by HPLC under optimized chromatographic conditions. The results showed that all samples showed a peak of similar shape and retention time as those of the CGA standard (Fig. 4). The retention time of authentic CGA was 13.376 min, and the retention times of the analyses ranged from 13.258 to 13.430 min at samples. There were relatively small differences in the maintained retention times among samples, and no interference was found (Fig. 4). The extracts from strains B14, B17, B19, N21, N22, P212, and S216 were revealed to contain CGA as a natural product according to the comparison of retention times and UV spectra of positive samples with those of authentic CGA (Fig. S3A). A significantly heightened peak was also observed at the same retention time when 0.1 μg authentic CGA was separately diluted with samples B14, B17, B19, N21, N22, P212, and S216 (Fig. S3B).

### Chlorogenic acid contents in the fermentation extracts of selected strains, assessed using Ultra performance liquid chromatography-mass spectrometry analysis

**Figure 5:**
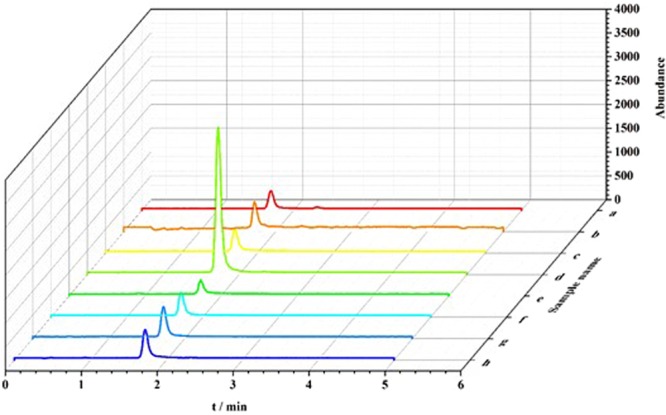
Typical multiple reaction monitoring chromatograms of chlorogenic acid (**a**) and extracts from strain B1-4 (**b**) strain B1-7 (**c**) strain B1-9 (**d**) strain N2-1 (**e**) strain N2-2 (**f**) strain P2-1-2 (**g**) and strain S2-16 (**h**).

The extracts from strains B14, B17, B19, N21, N22, P212, and S216 were analyzed using UPLC-MS. To provide a basis for the diagnostic identification of CGA in the extracts from the strains, authentic CGA was evaluated to ascertain the MS/MS fragmentation pattern. The production of abundant negative [M-H]^−^ ions and MS/MS fragmentation of authentic CGA could be easily detected at *m/z* 353.0876 (Fig. S4-1A) and 191.0552 (Fig. S4-1B), respectively. The products ions of [M-H]^−^ ions of the extracts of strain 216 were also confirmed at [M-H]^−^ *m/z* 353.0877 and 191.0553 (Fig. S4-1C,D) by LC-ESI-HRMS analysis. Other similar structural chemicals (caffeic acid, trans-Cinnamic acid, p-coumaric acid) were also checked, only caffeic acid was found in the product of strain S216, the detection of production ion is at *m/z* 170.0339 (Fig. S4-2). All the crude extracts were analyzed quantitatively according to this established method for determining CGA by UPLC-MS (Fig. 5).

UPLC-MS analysis showed that the retention time of authentic CGA was 1.71 min (Table 3 and Fig. 5), and a standard curve of CGA was created to calculate the level of CGA in the samples (Fig. S5). The retention times of samples from strains were very similar that of the standard CGA, and the production of CGA ranged from 0.08 mg L^−1^ to 23.39 mg L^−1^ among the screened strains (Table 3). A 3D stereogram was created to easily view the differences between the CGA contents of the samples (Fig. 5).

**Table 3:** The genera of microbial strains identified to produce chlorogenic acid and analysis of the retention time, peak area, and chlorogenic acid yield from some CGA-positive strains using UPLC-MS.

Source materials	The genera of CGA-positive strains			CGA-positive strains using UPLC-MS		
	Strain number	Genus	The NCBI GeneBank accession number	Retention time (min)*	Peak area (m^2^)	Yield (mg L^−1^)
*Mentha*	B14	*Brevibacillus borstelensis*	MG552835	1.71	3032	0.08
	B17	*Bacillus amyloliquefaciens*	MG552836	1.71	73196	1.75
	B111	*Bacillus subtilis*	MG552852	—	—	—
	B19	*Bacillus badius*	MG552833	1.72	227918	5.43
*Ipomoea batatas*	N21	*Sphingomonas yabuuchiae*	MG552834	1.74	547633	13.04
	N22	*Enterobacter tabaci*	MG554640	1.71	71029	1.69
	P212	*Lodderomyces elongisporus*	MG554644	1.72	73876	1.76
	N23	*Paenibacillus phoenicis*	MG554641	—	—	—
*Camellia*	S216	*Colletotrichum acutatum*	MG553373	1.72	980079	23.39
*Lonicera japonica*	N224	*Enterobacter tabaci*	MG554645	—	—	—
*Lonicera hypoglauca*	P292	*Lodderomyces elongisporus*	MG554642	—	—	—
	D215	*Colletotrichum acutatum*	MG554643	—	—	—
*Eucommia*	P2102	*Lodderomyces elongisporus*	MG554647	—	—	—

Note: A short dash (—) indicates that the strains are not checked using UPLC-MS method.

16S rDNA/18S rDNA sequence of B14, B17, B19, B111, N21, N22, N23, P212, N224, P292, D215, P2102 and S216 were compared with those in the NCBI database, which were separately identified with high similarity to *Brevibacillus borstelensis* (99%), *Bacillus amyloliquefaciens* (99%), *Bacillus subtilis* (99%), *Bacillus badius* (99%), *Sphingomonas yabuuchiae* (99%), *Enterobacter tabaci* (99%), *Paenibacillus phoenicis* (99%), *Lodderomyces elongisporus* (100%), *Enterobacter tabaci* (99%), *Lodderomyces elongisporus* (100%), *Colletotrichum acutatum* (99%), *Lodderomyces elongisporus* (100%), and *Colletotrichum acutatum* (99%) (Table 3, Supporting Information 1). The percentages in brackets represent the similarity to the sequences deposited in the NCBI database.

## Discussion

### The design of modified media to isolate chlorogenic acid-producing microbes

CGA could markedly inhibit tumor promotion and protect against lipid peroxidation^3,22^. The isolation of bioactive components from endophytic strains has been considered an alternative avenue to develop bioactive agents^23^. The chemical structure of CGA allows it to combine with Al^3+^ at pH values of 6.0–7.5^20^, producing a stable red-violet complex in both liquid and solid states. The complex formation and its color development showed no differences between the liquid BEA (pH = 6.0) and PDA (pH = 7.2–7.4) media at a low concentration of aluminum (III) (0.375 and 0.75 mM); however, the addition of a high concentration of aluminum (III) affected the solid state of BEA and PDA. Thus, the modified medium supplemented with 0.375 and 0.75 mM aluminum (III) was optimal for producing solid medium, and the color development was faster on the modified medium containing 0.75 mM aluminum, as assessed by the diameters of the color halos. In addition, the rate of color formation or the variation in the diameter of the halo among the isolated strains would likely be related to their biosynthetic capacity for CGA.

Here, a protocol was provided to isolate CGA-producing strains, comprising: (i) collecting the environmental samples; (ii) preparing modified media; (iii) smearing the sample over a screening plate containing the modified medium and returning the plate to the 37 °C or 28 °C incubator; (iv) isolating strains that showed color development on the modified media; (v) confirming and quantifying the level of CGA for the strains producing a color halo by HPLC or UPLC analysis.

### Comparison of the selection efficiency between traditional and modified methods

Many studies have shown that a series of valuable secondary products can be derived from endophytes, and this has also been considered as a prospective approach to produce polyphenol compounds like CGA^24,25^. Although many strains of bacteria or fungi were isolated from leaves, fruits, and other tissues of plants using basal media, few could produce CGA, as assessed by HPLC^19^. Therefore, it is relatively difficult to isolate CGA-producing strains in a high-throughput manner using a traditional screening method. However, the present study presents a new method to screen CGA-producing strains, based on modified media. HPLC showed that 10 of 11 strains from the modified media produced CGA (90.9%); whereas, an efficiency of 2.8% was obtained strains derived from the basal media. It appeared that the media with aluminum ions was actually selective to detect CGA-producing strains, probably because the media with aluminum ions were specific for CGA in the range of pH 6.0–7.4, exhibiting maximum absorbance value at 570 nm. Whereas, the formation of complex with other polyphenolic compounds (rutin and quercetin) varied in color development and maximum absorbance values, which was consistent with the maximum absorbance values of other phenolic compounds (rutin, quercetin and catechin) at 510 nm (or 410–430 nm)^26–28^.

The modified medium possessed three important characteristics. First, it was simple to make, and only 0.75 mM aluminum (III) needed to be added to the basal media. Second, it was highly efficient for isolating strains with the capacity to produce CGA, identifying approximately 3.3 times more CGA producers than the conventional method. Third, it was less expensive compared to the cost of isolating strains on the basal media, because a lower total number of strains grew on the modified media compared with those on the basal media, and the color development could be used to judge whether the strains could produce CGA. HPLC analysis showed that most strains isolated from the basal media did not have the ability to produce CGA, and few of them showed a color change on the modified media; thus, the expense of further analyses and unnecessary labor could be saved. Therefore, this method is a simple, precise, and inexpensive process to isolate CGA-producing strains. The design is compatible with high-throughput plating isolation in a time- and labor-efficient manner.

### Application of modified medium

Improving the isolation of microbes that synthesize CGA may require a more radical approach than the traditional methods. The screening method presented in this study was based on the molecular coupling of CGA with Al^3+^ following reports^20,21^, and was firstly found to be highly efficient in isolating CGA-producing microbes. Whereas the strains that did not develop a color on the modified medium were confirmed to not produce CGA. The present study indicated that the selection method represents a powerful tool to screen microbes that can synthesize polyphenol compounds from plants and soil, adding the possibility that this method is applicated to quantify the CGA of plants or isolate a new species of plants.

CGA is not a common trait in all plants, but it is often presence in most medical plants (*Mentha*, *Eucommia ulmoides*, *Lonicera hypoglauca*, *Ipomoea batatas*, *Camellia* and so on), and it is also abundant in fruits and vegetables, such as coffee beans, Mango, apples^29–31^. Plant endophytes could colonize the internal tissues in nearly every plant. Endophytes in symbiosis with CGA-producing plants could produce chlorogenic acid probably because of the existence of similar secondary metabolic pathway. In the present study, most of positive microbial species were firstly reported as CGA producers, including *Brevibacillus borstelensis* sp. B14, *Bacillus amyloliquefaciens* sp. B17, *Bacillus badius* sp. B19, *Sphingomonas yabuuchiae* sp. N21, *Enterobacter tabaci* sp. N22, *Lodderomyces elongisporus* sp. P212, and *Lodderomyces elongisporus* sp. S216. Among the identified CGA-synthesizing microbes, the CGA yield of strain S216 was the highest, reaching 23.39 mg L^−1^ before optimizing the fermentation conditions, and two other strains, *Sphingomonas yabuuchiae* sp. N21 and *Bacillus badius* sp. B19, produced CGA at 13.04 and 5.43 mg L^−1^, respectively. Although the CGA yield of the strains were low, they could be improved by optimizing the fermentation conditions and regulating key enzymes involved in CGA biosynthesis to obtain a higher quantity of CGA^19^.

In summary, we studied CGA-producing strains isolated from the environment and developed a novel method to isolate CGA-producing microbes from plants, soil, and other environments. This screening method is rapid, accurate, inexpensive, and compatible with high-throughput plating isolation. We also identified more than a dozen newly isolated strains that could be used for CGA production.

## Materials and Methods

### Plant materials

To isolate strains that produce CGA, fresh leaves were collected from *Ipomoea batatas*, *Eucommia ulmoides Oliver*, *Lonicera japonica*, *Camellia*, *Lonicera hypoglauca*, *Metha haplocalyx*, and *Laurocerasus phaeosticta*, during March of 2017 at Guangxi University and Guangxi Medicinal Botanical Garden (N22°50′28.41″, E108°17′9.00″), Nanning, Guangxi Province, China. Samples were individually packed into sterile plastic bags, dependent on the species, and stored at 4 °C.

### Drugs and Reagents

Authentic CGA (C_16_H_18_O_9_, CAS # 327-97-9, HPLC ≥98%), for use as a standard, was obtained from Shanghai Yuanye Biological Technology Co., Ltd (Shanghai, China). Chromatography-grade acetonitrile and methanol were purchased from the Tianjin Branch of the United States and Europe Chemical Reagent Co., Ltd. (Tianjin, China). Analytical-grade ethyl acetate, ethanol, acetic acid, formic acid, toluene iron (III) chloride hexahydrate (FeCl_3_·6H_2_O), and aluminum (III) chloride hexahydrate (AlCl_3_·6H_2_O) were supplied by Tianjin Fuyu Fine Chemical Co., Ltd. (Tianjin, China). All other chemicals were of reagent grade and were obtained from Sangon Biotech Co., Ltd (Shanghai, China).

### Constituents of basal medium and modified medium

Modified medium of microbes was prepared based on the stability of aluminum (III) complexes with CGA at pH 6.0–7.7 and chromogenic-complexing reaction reported by Adams *et al*.^20^. Beef Extract Agar (BEA, Basal medium) was prepared for the primary isolation of bacteria, as described previously^32^, with a slightly modified composition: Beef extract 3 g; peptone, 10 g; sodium chloride, 5 g; agar, 20 g; and distilled water to 1000 mL. The pH of medium after autoclaving was 7.2–7.4. The modified BEA medium was prepared to isolate CGA-biosynthesizing bacteria using the above composition with the addition of 0.375–0.75 mM aluminum chloride.

Potato Dextrose Agar (PDA, Basal medium) was used to culture fungi with the following composition: Potato extract, 300 g; dextrose, 20 g; chloramphenicol, 0.1 g; agar, 20 g; pH 6.0–70. The PDA medium was supplemented with 0.375–0.75 mM aluminum chloride (modified medium) to identify the CGA-biosynthesizing fungi. Liquid medium was also prepared using the above ingredients but without agar. All media used for screening were sterilized by autoclaving (121 °C, 15 min).

### Isolation of endophytic bacterial and endophytic fungi

The general procedures adopted for isolation of the microorganisms were based on the methods described by Weber *et al*.^33^. To ensure that the leaf surfaces were sterilized completely and to screen endophytic strains from plants, samples of plant leaves were soaked in sterile flask containing 75% alcohol for 60 s in a laminar flow hood, and subsequently with 5.0% sodium hypochlorite for 15 s after washing off the alcohol residue with sterile water. To isolate CGA-producing bacteria, 1-cm^2^ wide pieces were cut from the disinfected leaf surface and then inoculated onto modified BEA medium. The cultures were examined for color development after 5 d of culture at 37 °C (in the dark). The stable complexes formed between CGA and aluminum chloride produced a violet-red color. Similar inoculation and cultivation were carried out on modified PDA plates to select CGA-biosynthesizing fungi at 28 °C (in the dark). The isolates with a violet-red circle on the medium were selected, cultured individually, and stored as a glycerol stock at −80 °C for further analysis. Conventional isolation was also performed on basal medium, and all isolates were examined for their ability to produce CGA using high-performance liquid chromatography (HPLC) analysis after cultivation in parallel liquid basal medium.

All the strains isolated from basal media and modified media as described in above section were stored at 4 °C. Each isolated strain was separately inoculated into a 250 mL Erlenmeyer flask containing 100 mL of parallel liquid medium (BEA or PDA medium without agar). The bacterial strains were fermented with shaking at 200 rpm at 37 °C for 1 d, and the fungal strains were fermented for 5 d at 28 °C and 140 rpm. After fermentation, the products from each strain were used to evaluate the potential of the strain to produce CGA.

First, 15 mL of fermentation liquid was sampled from the Erlenmeyer flasks into a 50 mL plastic centrifuge tube, and the pH was adjusted to 5.0 using 1 M hydrochloric acid. Subsequently, the bacteria in the centrifuge tube were subjected to 30 min of ultrasonic disruption to release the cell contents, and then centrifuged at 7100 × *g* for 10 min. Then, 1 mL of the supernatant was removed and added to 1 mL of 70% ethanol, and the mixture was allowed to stand for 5 h. The mixture was concentrated under a vacuum at 45 °C for 30 min until dry, and then the same volume of ethyl acetate was added for further extraction; this process was repeated three times. The ethyl acetate layer was concentrated until it was almost dry, and finally, 1 mL of methanol was added to the tube to completely dissolve the extraction products. The methanol extract was tested for its level of CGA using thin layer chromatography (TLC) and HPLC.

### Thin layer chromatography

The contents of each methanol extract were determined qualitatively using TLC, following the selective method reported by Tao and Yang^34^. TLC sheets were cut to 10 × 10 cm, and TLC plate start lines were drawn using a pencil at 1.0 cm from the bottom. Extracts were each dissolved in 1 mL methanol carefully spotted onto the line using a capillary tube, and 1 mg L^−1^ of the CGA standard was spotted as a control. Chromatograms were developed in a normal chromatographic chamber that was pre-saturated with the mobile phase (ethyl acetate:water:formic acid:toluene (80:10:9:5 v/v/v/v)^35^. TLC was run until the mobile phase reached about 2 cm from the upper edge. After the plate was air dried, significant blue spots (representing CGA) could be detected visually by spraying the plate with a mixture of 1% potassium ferricyanide and 1% ferric chloride solutions. Finally, the retardation factor (R_f_) value of CGA was measured following the identification of the blue spots^36^.

### High-performance liquid chromatography analysis

The previously prepared extracts of each strain were separately condensed with methanol and further condensed with an equal volume of ethyl acetate at 45 °C for 30 min. Subsequently, the mixture was centrifuged at 16000 × *g* for 10 min^35^, and 10 L of the supernatant was subjected to HPLC analysis.

HPLC was conducted using an LC-6AD system, and a chromatographic column (JADE-PAK ODS-AQ C-18 stainless steel; 250 mm × 4.6 mm, 5 μm; Echway Corporation, Guangzhou, China) was used with a mobile phase of 0.5% acetic acid:acetonitrile (92:8). The flow rate was set to 1.0 mL min^−1^, and the temperature of the column was maintained at 35 °C. UV detection was performed at 327 nm.

### Ultra performance liquid chromatography-mass spectrometry analysis of the extract of the selected strain

Ultra performance liquid chromatography (UPLC) was performed using an Agilent Technologies Co. (Agilent, Santa Clara, USA) system equipped with a binary solvent delivery system, an auto-sampler, and a photo-diode array detector^19^. Chromatography was performed on an Rrhd-Zorbax C_18_ column (2.1 × 50 mm, 1.8 μm) with a gradient elution of 90% A (0.5% acetic acid in water) and 10% B (acetonitrile) (0–5 min); 75% A and 25% B (5–6 min); and 90% A and 10% B (6–9 min) at 25 °C. The auto-sampler was set to 1 μL and the flow rate was 0.3 mL min^−1^. The UPLC/MS analysis was performed using an electrospray ionization source (ESI) and a detecting mode of multiple reactions monitoring (MRM). Mass calibration and resolution adjustments were performed on the UPLC using an infusion of formate acid before the mass spectrometry (MS) experiments^37^. In the analysis, a Lock MS mode was applied for the experiment to calibrate the molecular weight in real time using authentic CGA (353.08493). The ESI was operated in negative ion mode, with a spray voltage of 4 kV. The optimal MS parameters were as follows: Capillary voltage, 4 kV (ESI+), 3 kV (ESI−); nozzle voltage, 0 V; spray voltage, 4 kV; and nitrogen was used as the nebulizer gas at a pressure of approximately 2.8 × 10^5^ Pa. The nitrogen flow was 600 L h^−1^ and 720 L h^−1^ for the desolvation and sheath gases, respectively. The desolvation gas was heated to 300 °C, and the temperature of the sheath gas was set at 360 °C. Highly pure nitrogen was used as the collision gas.

### Polymerase chain reaction amplification of 16S rDNA, 18S rDNA, and DNA sequencing

The bacterial and fungal DNA were separately extracted from fresh CGA-positive colonies grown on parallel modified medium using a Takara MiniBEST Bacteria Genomic DNA Extraction Kit or a Tiangen plant genomic extraction kit. The gene encoding the 16S rDNA was amplified by polymerase chain reaction (PCR) using universal primers 27F (5′-GAGTTTGATCCTGGCTCAG-3′) and 1492R (5′-ACCTTGTTACGACTT-3′), as described previously^38^, and the gene encoding 18S rDNA was amplified by PCR using a forward primer (EP3):5′-GGAAGGGRTGTATTTATTAG-3′ and reverse primer (EP4): 5′-TCCTCTAAATGACCAAGTTTG-3′, as reported by Mishra *et al*.^39^. The PCR products were purified and sequenced by an external laboratory (BGI-Shenzhen, Shenzhen City, China). The nucleotide sequences were compared with the 16S or 18S rDNA sequences in the NCBI database using BLASTN, and the closest match with a known phylogenetic affiliation was used to assign the isolated strains to specific taxonomic groups.

### Measurement of absorption peaks and color development of chlorogenic acid-Al^3+^ complexes at different pH values

CGA, which is an organic carboxylic acid with phenolic hydroxyl groups, can be coupled with aluminum ions. The chromogenic reaction between CGA and aluminum (III) was studied at a ratio of 2:1, as described previously^20^, with a slight modification. Briefly, CGA and AlCl_3_ were dissolved in 5 mL of liquid BEA medium to produce a 1.8 mM (CGA) and 0.9 mM (AlCl_3_) solution. The mixture was then separately adjusted to pH 6.5, 7.0, and 7.5 using 1 M NaOH solution. The same compositions were dissolved in 5 mL distilled water as controls. After 30 min incubation at room temperature, the mixture was subjected to spectral analysis in the range of 450 to 700 nm against a blank in which the AlCl_3_ solution was substituted by water.

## Electronic supplementary material

Supporting Information
