# Supplementary material for: A novel design to screen chlorogenic acid-producing microbial strains from the environment
Source: Sci Rep. 2018 Oct 3;8:14756. doi: 10.1038/s41598-018-32968-0 (PMC6170458; doi:10.1038/s41598-018-32968-0)
Supplement: Supplementary file 1 — Supporting Information [file 41598_2018_32968_MOESM1_ESM.pdf]

Title Page

Title: A novel design to screen chlorogenic acid-producing microbial strains from the environment

Xiao Wang [1], Lifang Qin [1], Jia Zhou [1], Youzhi Li [1] *, Xianwei Fan [1] *

[1] State Key Laboratory for Conservation and Utilization of Agricultural Bioresource in Subtropics, College of Life Science and Technology, Guangxi University, Nanning 530004, China

*Correspondence and requests for materials should be addressed to F.X.W (email: fanxw@gxu.edu.cn) or L.Y.Z. (email: dyzl@gxu.edu.cn)

Tel: +86-771-3272130

Fax: +86-771-3272130

Table S1. Effect of culture time on the halo diameter (mm, means ± se.) of strain on the medium with Al-free, low and high Al

Medium	Day			
	5 d	6 d	7 d	8 d
Al-free	1.50 ± 0.01	2.00 ± 0.04	3.73 ± 0.07	4.93 ± 0.03
Low Al	3.26 ± 0.02	3.82 ± 0.02	5.02 ± 0.02	5.40 ± 0.01
High Al	4.22 ± 0.03	4.48 ± 0.01	5.56 ± 0.01	6.50 ± 0.01

Table S2 Comparison of the retardation factor (R_{f}) of the fermentation products of each isolated strain with that of chlorogenic acid (CGA)

Strain number	CGA	P212	P292	P2102	B14	B17	B19	S216
R_f	0.75 ±0.01	0.74 ±0.02	0.73 ±0.01	0.75 ±0.01	0.74 ±0.01	0.74 ±0.01	0.77 ±0.03	0.75 ±0.07
Strain number	N22	N21	D215	N222	N25	N231	N239	N32
R_f	0.74 ±0.02	0.75 ±0.01	0.75 ±0.01	0.77 ±0.01	0.82 ±0.02	0.79 ±0.04	0.82 ±0.03	0.70 ±0.01
Strain number	CGA	N24	N220	D230	N31	N23	B111	N224
R_f	0.75 ±0.01	0.71 ±0.01	0.72 ±0.02	0.70 ±0.03	0.71 ±0.02	0.74 ±0.03	0.76 ±0.02	0.74 ±0.04

Supplementary Figures

To check the chelating ability of other similar structural compounds with Al^{3+}, shikimic acid, quinine acid, caffeic acid, trans-cinnamic acid, p-coumaric acid and 5-O-caffeoylshikimic acid were chosen and respectively, dissolved in 15 ml tubes with 5 mL of liquid BEA medium to produce a 1.8 mM above structural compounds (shikimic acid quinine acid, caffeic acid, trans-cinnamic acid, p-coumaric acid and 5-O-caffeoylshikimic acid), then $AlCl_3$ was added in two tubes to produce 0.9 mM $AlCl_3$ solution. Subsequently, the mixture was then separately adjusted to pH 7.0 using 1 M NaOH solution. After 30 min incubation at room temperature, the mixture was subjected to spectral analysis in the range of 200 to 800 nm. CGA were also carried out as a control. The results showed that no absorbance appeared at 400-800 nm in the mixture solution of shikimic acid and $AlCl_3$ (Figure S1. A), and similar results occurred in the mixture solution of other similar structural chemicals (quinine acid, caffeic acid, trans-cinnamic acid, p-coumaric acid and 5-O-caffeoylshikimic acid) and $AlCl_3$ (Figure S1. B-F). However, a single absorption peak was observed at 570 nm after formation of the CGA-Al^{3+} complex at pH 7.0 (Figure S1. G). In addition, the purple color development of mixture solution was only observed in the mixture solution of CGA and $AlCl_3$ (Figure S1. G). Whereas, the color development appeared yellow in the mixture solution of 5-O-caffeoylshikimic acid and $AlCl_3$ (Figure S1. F). In all, these results indicated that the proposed method is specific for CGA and confers to detect CGA-synthetic strains quickly.

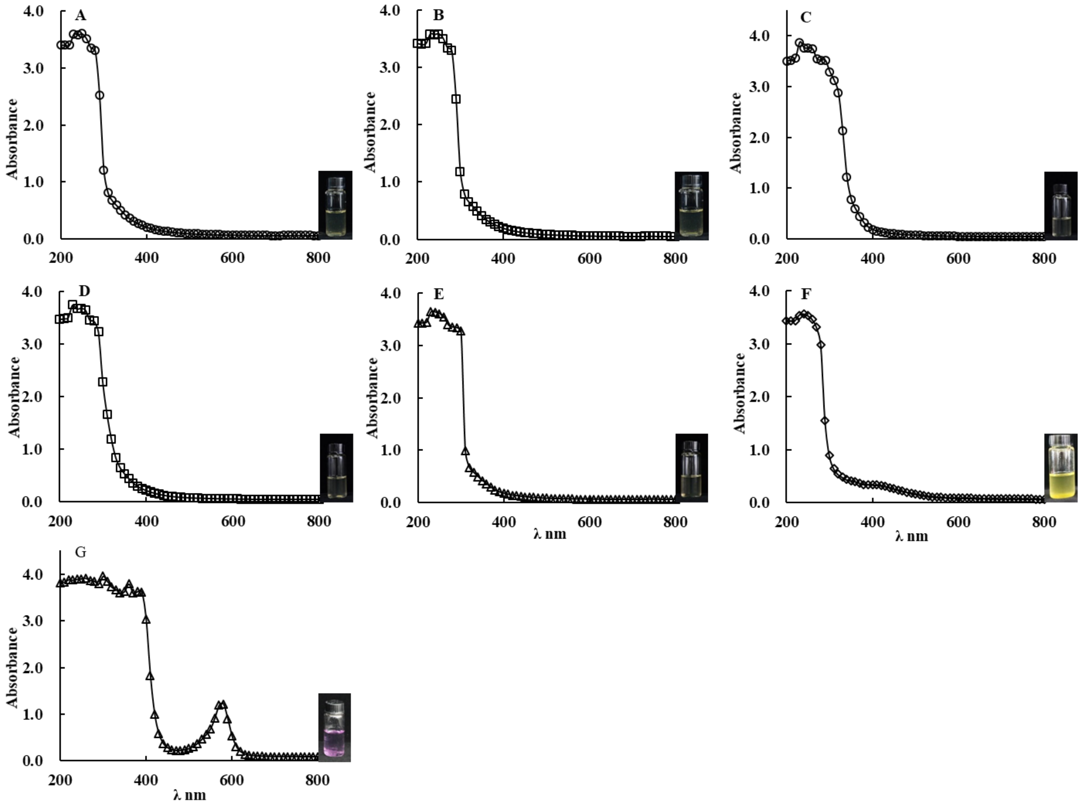

Figure S1. Comparison of the absorption spectra and color development of the complex formed between shikimic acid (A), quinine acid (B), Caffeic acid (C), trans- Cinnamic acid (D), p-coumaric acid (E), 5-O-caffeoylshikimic acid (F) and chlorogenic acid (G) and aluminum (III) in beef extract agar (BEA) liquid medium. [Al] = 0.9 mM, [chlorogenic acid] = 1.8 mM, [shikimic acid] = 1.8 mM, 5-O-caffeoylshikimic acid = 1.8 mM, [quinine acid] = 1.8 mM, [Caffeic acid] = 1.8 mM, [trans- Cinnamic acid] = 1.8 mM and [p-coumaric] = 1.8 mM.

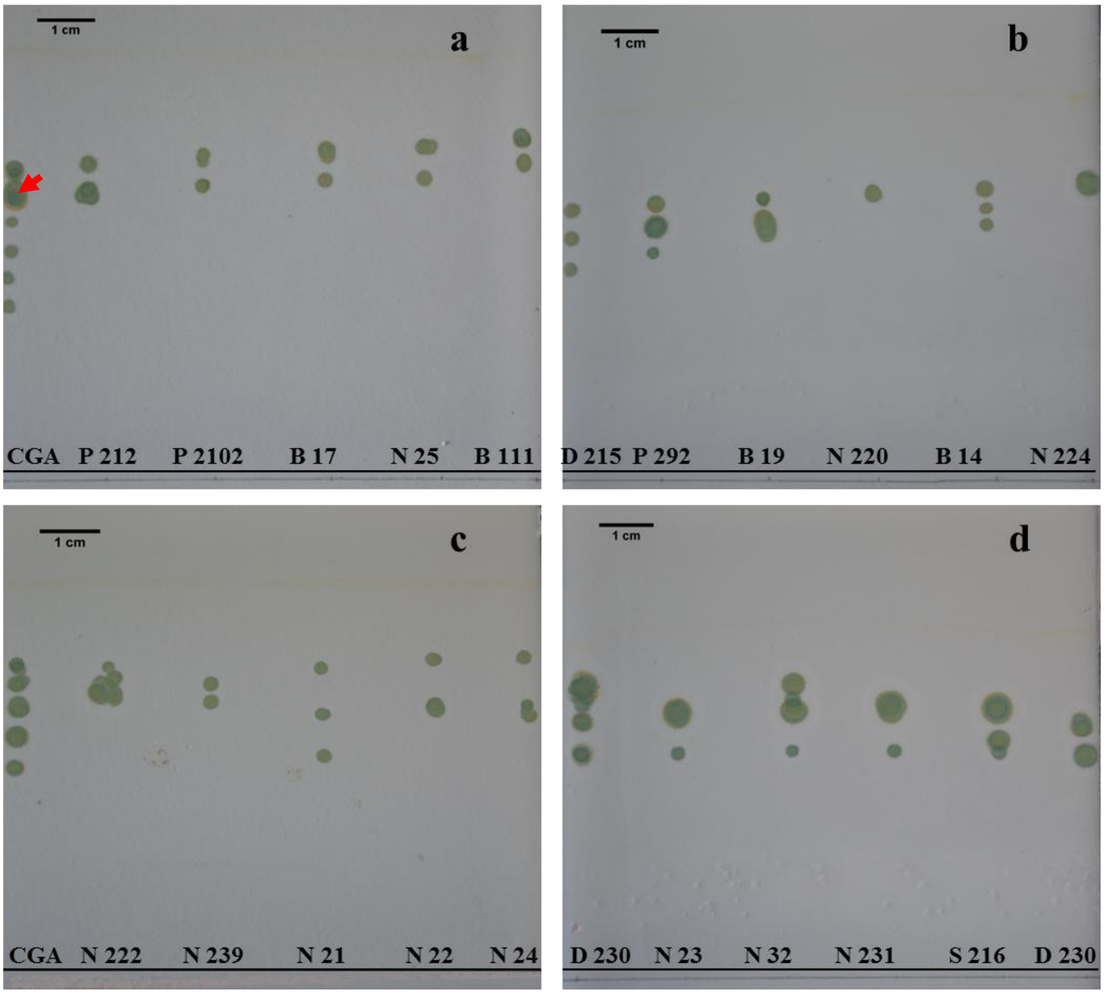

Figure. S2 Thin Layer Chromatography of authentic chlorogenic acid (CGA) and the extracts of isolated strains. Red arrowhead indicates the pure spot of CGA standard sample.

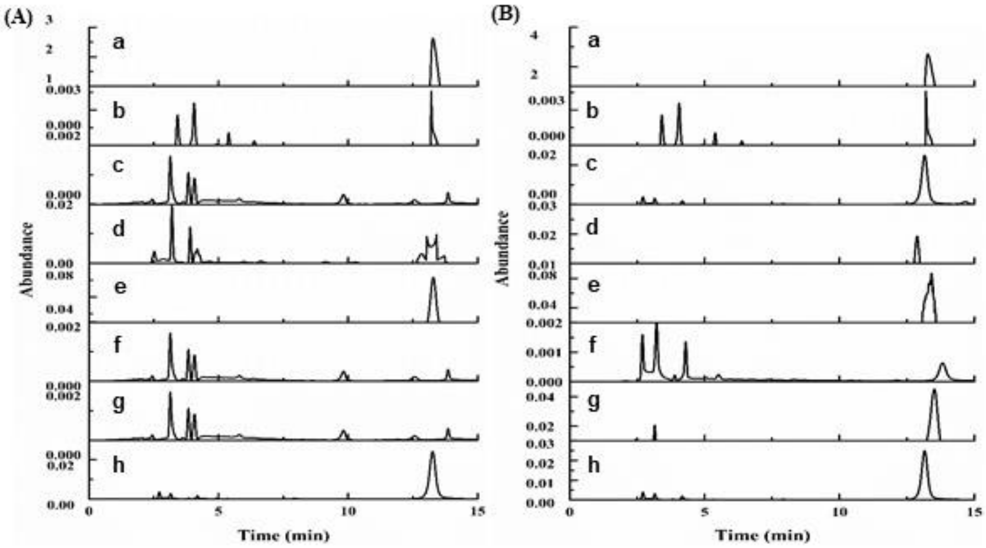

Figure. S3 Comparative analysis of the retention times of chlorogenic acid in the extracts of different strains. A: the individual extracts of the strain samples, S216 (a), P212 (b), N22 (c), N21 (d), B19 (e), B17 (f), B14 (g); B: the individual extracts of the strains examples was mixed with 2 μg authentic chlorogenic acid (h).

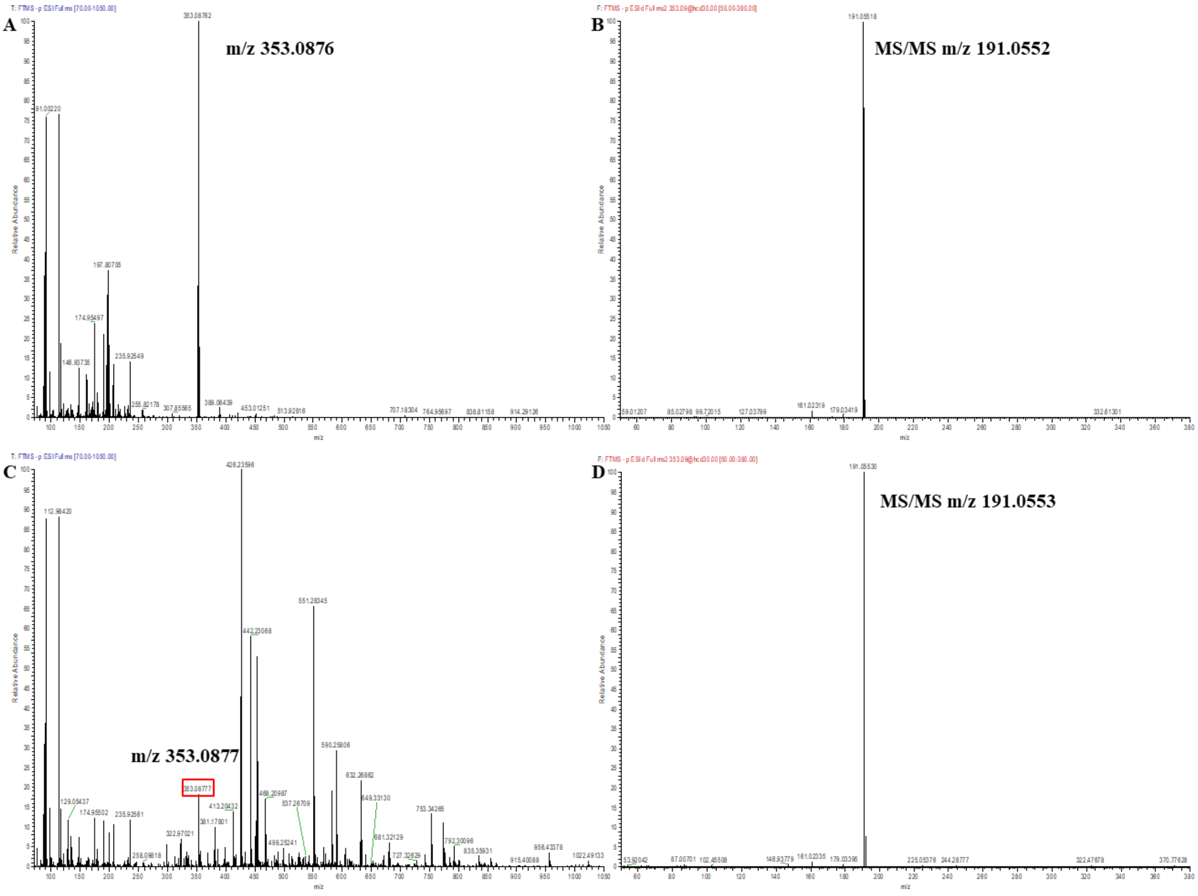

Figure. S4-1 Mass spectrometry (MS) and MS/MS characterization of 0.2 mg L^{-1} authentic chlorogenic acid (A, B) and those of fermentation product of strain S216 (C, D). (Thermo Q Exactive, Massachusetts, USA)

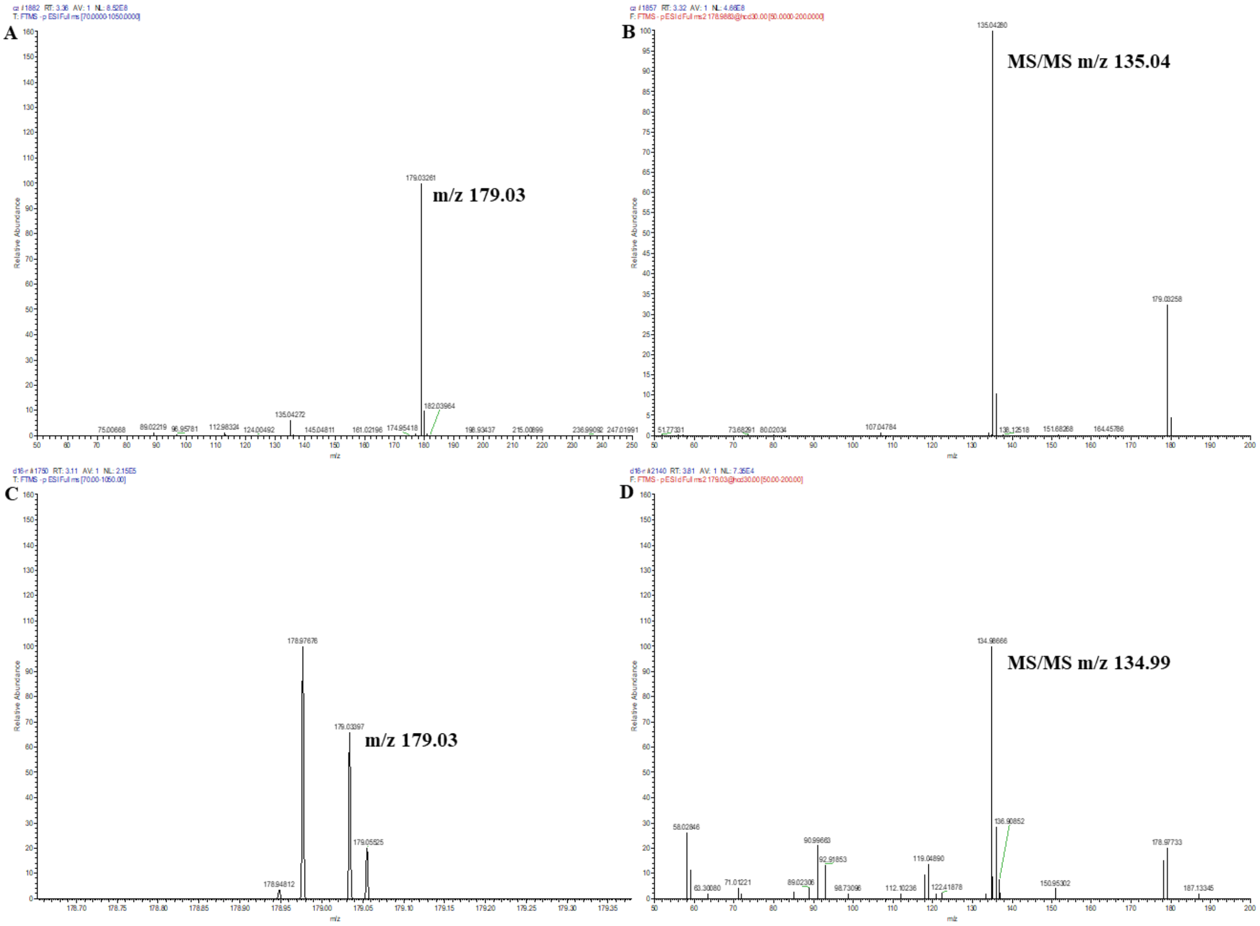

Figure S4-2 Mass spectrometry (MS) and MS/MS characterization of 0.2 mg L^{-1} authentic Caffeic acid (A, B) and those of fermentation product of strain S216 (C, D). (Thermo Q Exactive, Massachusetts, USA)

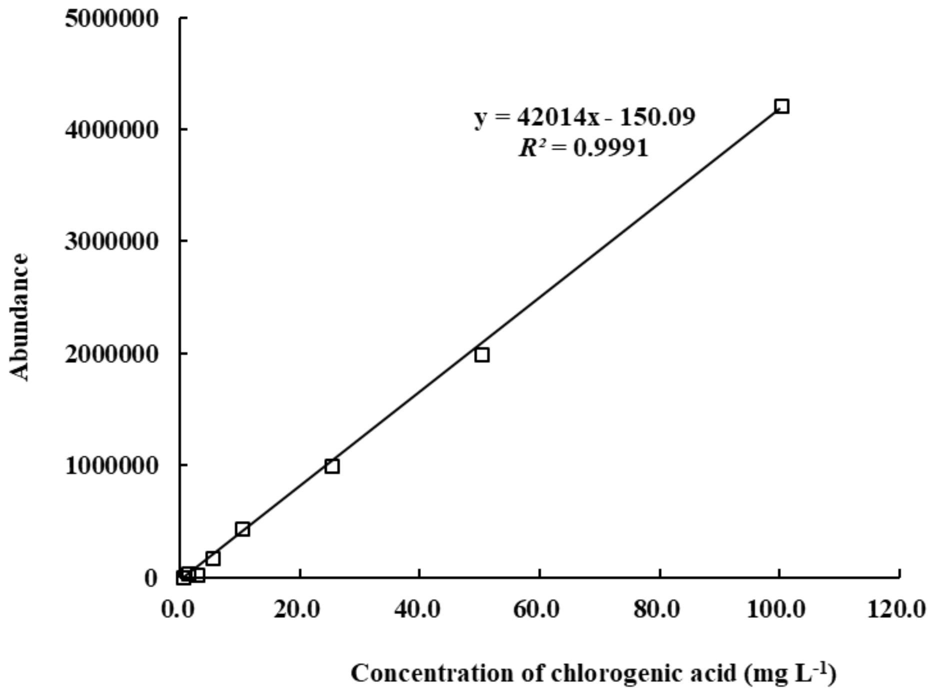

Figure. S5 Calibration curve for UPLC-MS with chlorogenic acid standard solution of 0-100 mg L^{-1}

Supporting Information 1

The sequence of the biosynthetic CGA strains

B14:

GGGGGGGGACCGTCTAATAATGCAGTCGAGCGAGTCCCTTCGGGGGCTAGCGGCGGACGGGTGAGTAACACGTAGGCAA
CCTGCCCGTAAGCTCGGGATAACATGGGGAAACTCATGCTAATACCGGATAGGGTCTTCTCTCGCATGAGGGGAGACGGA
AAGGTGGCGCAAGCTACCACTTACGGATGGGCCTGCGGCGCATTAGCTAGTTGGTGGGGTAACGGCCTACCAAGGCGACG
ATGCGTAGCCGACCTGAGAGGGTGACCGGCCACACTGGGACTGAGACACGGCCCAGACTCCTACGGGAGGCAGCAGTAG
GGAATTTTCCACAATGGACGAAAGTCTGATGGAGCAACGCCGCGTGAACGATGAAGGTCTTCGGATTGTAAAGTTCTGTT
GTCAGAGACGAACAAGTACCGTTCGAACAGGGCGGTACCTTGACGGTACCTGACGAGAAAGCCACGGCTAACTACGTGCC
AGCAGCCGCGGTAATACGTAGGTGGCAAGCGTTGTCCGGAATTATTGGGCGTAAAGCGCGCGCAGGCGGCTATGTAAGTC
TGGTGTTAAAGCCCGGGGCTCAACCCCGGTTCGCATCGGAAACTGTGTAGCTTGAGTGCAGAAGAGGAAAGCGGTATTCC
ACGTGTAGCGGTGAAATGCGTAGAGATGTGGAGGAACACCAGTGGCGAAGGCGGCTTTCTGGTCTGTAACTGACGCTGAG
GCGCGAAAGCGTGGGGAGCAAACAGGATTAGATACCCTGGTAGTCCACGCCGTAAACGATGAGTGCTAGGTGTTGGGGGT
TTCAATACCCTCAGTGCCGCAGCTAACGCAATAAGCACTCCGCCTGGGGAGTACGCTCGCAAGAGTGAAACTCAAAGGAA
TTGACGGGGGCCCGCACAAGCGGTGGAGCATGTGGTTTAATTCGAAGCAACGCGAAGAACCTTACCAGGTCTTGACATCC
CGCTGACCGTCCTAGAGATAGGGCTTCCCTTCGGGGCAGCGGTGACAGGTGGTGCATGGTTGTCGTCAGCTCGTGTCGTGA
GATGTTGGGTTAAGTCCCGCAACGAGCGCAACCCTTATCTTTAGTTGCCAGCATTCAGTTGGGCACTCTAGAGAGACTGCC
GTCGACAAGACGGAGGAAGGCGGGGATGACGTCAAATCATCATGCCCCTTATGACCTGGGCTACACACGTGCTACAATGG
CTGGTACAACGGGAAGCTAGCTCGCGAGAGTATGCCAATCTCTTAAAACCAGTCTCAGTTCGGATTGCAGGCTGCAACTCG
CCTGCATGAAGTCGGAATCGCTAGTAATCGCGGATCAGCATGCCGCGGTGAATACGTTCCCGGGCCTTGTACACACCGCCC
GTCACACCACGGGAGTTTGCAACACCCGAAGTCGGTGAGGTAACCGCAAGGAGCCAGCCGCCGTAAGTTGTAACCT

B17:

ATGGTTCAAATTATACCCGCTATAGTGTGAGTCGAGCGGACAGATGGGAGCTTGCTCCCTGATGTTAGCGGCGGACGGGT
GAGTAACACGTGGGTAACCTGCCTGTAAGACTGGGATAACTCCGGGAAACCGGGGCTAATACCGGATGGTTGTCTGAACC
GCATGGTTCAGACATAAAAGGTGGCTTCGGCTACCACTTACAGATGGACCCGCGGCGCATTAGCTAGTTGGTGAGGTAAC
GGCTCACCAAGGCGACGATGCGTAGCCGACCTGAGAGGGTGATCGGCCACACTGGGACTGAGACACGGCCCAGACTCCTA
CGGGAGGCAGCAGTAGGGAATCTTCCGCAATGGACGAAAGTCTGACGGAGCAACGCCGCGTGAGTGATGAAGGTTTTCGG
ATCGTAAAGCTCTGTTGTTAGGGAAGAACAAGTGCCGTTCAAATAGGGCGGCACCTTGACGGTACCTAACCAGAAAGCCA
CGGCTAACTACGTGCCAGCAGCCGCGGTAATACGTAGGTGGCAAGCGTTGTCCGGAATTATTGGGCGTAAAGGGCTCGCA
GGCGGTTTCTTAAGTCTGATGTGAAAGCCCCCGGCTCAACCGGGGAGGGTCATTGGAAACTGGGGAACTTGAGTGCAGAA
GAGGAGAGTGGAATTCCACGTGTAGCGGTGAAATGCGTAGAGATGTGGAGGAACACCAGTGGCGAAGGCGACTCTCTGG
TCTGTAACTGACGCTGAGGAGCGAAAGCGTGGGGAGCGAACAGGATTAGATACCCTGGTAGTCCACGCCGTAAACGATGA
GTGCTAAGTGTTAGGGGGTTTCCGCCCCTTAGTGCTGCAGCTAACGCATTAAGCACTCCGCCTGGGGAGTACGGTCGCAAG
ACTGAAACTCAAAGGAATTGACGGGGGCCCGCACAAGCGGTGGAGCATGTGGTTTAATTCGAAGCAACGCGAAGAACCTT
ACCAGGTCTTGACATCCTCTGACAATCCTAGAGATAGGACGTCCCCTTCGGGGGCAGAGTGACAGGTGGTGCATGGTTGTC
GTCAGCTCGTGTCGTGAGATGTTGGGTTAAGTCCCGCAACGAGCGCAACCCTTGATCTTAGTTGCCAGCATTCAGTTGGGC
ACTCTAAGGTGACTGCCGGTGACAAACCGGAGGAAGGTGGGGATGACGTCAAATCATCATGCCCCTTATGACCTGGGCTA
CACACGTGCTACAATGGACAGAACAAAGGGCAGCGAAACCGCGAGGTTAAGCCAATCCCACAAATCTGTTCTCAGTTCGG
ATCGCAGTCTGCAACTCGACTGCGTGAAGCTGGAATCGCTAGTAATCGCGGATCAGCATGCCGCGGTGAATACGTTCCCG
GGCCTTGTACACACCGCCCGTCACACCACGAGAGTTTGTAACACCCGAAGTCGGTGAGGTAACCTTTATGGAGACAGCCG
CCGTACAAGTGATAAGGCTGGG

B111:

GGCATGGGGATACTCTAGAGTGAGATCGTGAGCTCGAAGATGGGAGCTTGGTCCCTGAAGTTAGCGGCGGACGGGTGAGT
AACACGTGGGTAACCTGCCTGTAAGACTGGGATAACTCCGGGAAACCGGGGCTAATACCGGATGGTTGTTTGAACCGCAT
GGTTCAAACATAAAAGGTGGCTTCGGCTACCACTTACAGATGGACCCGCGGCGCATTAGCTAGTTGGTGAGGTAACGGCT
CACCAAGGCAACGATGCGTAGCCGACCTGAGAGGGTGATCGGCCACACTGGGACTGAGACACGGCCCAGACTCCTACGG
GAGGCAGCAGTAGGGAATCTTCCGCAATGGACGAAAGTCTGACGGAGCAACGCCGCGTGAGTGATGAAGGTTTTCGGATC
GTAAAGCTCTGTTGTTAGGGAAGAACAAGTACCGTTCGAATAGGGCGGTACCTTGACGGTACCTAACCAGAAAGCCACGG
CTAACTACGTGCCAGCAGCCGCGGTAATACGTAGGTGGCAAGCGTTGTCCGGAATTATTGGGCGTAAAGGGCTCGCAGGC
GGTTTCTTAAGTCTGATGTGAAAGCCCCCGGCTCAACCGGGGAGGGTCATTGGAAACTGGGGAACTTGAGTGCAGAAGAG
GAGAGTGGAATTCCACGTGTAGCGGTGAAATGCGTAGAGATGTGGAGGAACACCAGTGGCGAAGGCGACTCTCTGGTCTG
TAACTGACGCTGAGGAGCGAAAGCGTGGGGAGCGAACAGGATTAGATACCCTGGTAGTCCACGCCGTAAACGATGAGTG
CTAAGTGTTAGGGGGTTTCCGCCCCTTAGTGCTGCAGCTAACGCATTAAGCACTCCGCCTGGGGAGTACGGTCGCAAGACT
GAAACTCAAAGGAATTGACGGGGGCCCGCACAAGCGGTGGAGCATGTGGTTTAATTCGAAGCAACGCGAAGAACCTTACC
AGGTCTTGACATCCTCTGACAATCCTAGAGATAGGACGTCCCCTTCGGGGGCAGAGTGACAGGTGGTGCATGGTTGTCGTC
AGCTCGTGTCGTGAGATGTTGGGTTAAGTCCCGCAACGAGCGCAACCCTTGATCTTAGTTGCCAGCATTCAGTTGGGCACT
CTAAGGTGACTGCCGGTGACAAACCGGAGGAAGGTGGGGATGACGTCAAATCATCATGCCCCTTATGACCTGGGCTACAC
ACGTGCTACAATGGACAGAACAAAGGGCAGCGAAACCGCGAGGTTAAGCCAATCCCACAAATCTGTTCTCAGTTCGGATC
GCAGTCTGCAACTCGACTGCGTGAAGCTGGAATCGCTAGTAATCGCGGATCAGCATGCCGCGGTGAATACGTTCCCGGGC
CTTGTACACACCGCCCGTCACACCACGAGAGTTTCCACACCGGAAGTCGGGGAGCTAACATATGAGGACACAGTATAACG
TAGAGTGTACCCCCC

B19:

GGGCAAGCGACCCGCCTAGAATGCAGTCGAGCGGACTTGACGGAAGCTTGCTTCCGTTCAAGTTAGCGGCGGACGGGTGA
GTAACACGTGGGTAACCTGCCTGTAAGACTGGGATAACTCCGGGAAACCGGGGCTAATACCGGATATTCTTTTTCTTCGCA
TGAAGAAGAATGGAAAGGCGGCTTTTAGCTGTCGCTTACAGATGGACCCGCGGCGCATTAGCTAGTTGGTGAGGTAACGG
CTCACCAAGGCAACGATGCGTAGCCGACCTGAGAGGGTGATCGGCCACACTGGGACTGAGACACGGCCCAGACTCCTACG
GGAGGCAGCAGTAGGGAATCTTCCGCAATGGACGAAAGTCTGACGGAGCAACGCCGCGTGAGTGAAGAAGGTTTTCGGA
TCGTAAAGCTCTGTTGTCAGGGAAGAACAAGTACGGAAGTAACTGTCCGTACCTTGACGGTACCTGACCAGAAAGCCACG
GCTAACTACGTGCCAGCAGCCGCGGTAATACGTAGGTGGCAAGCGTTGTCCGGAATTATTGGGCGTAAAGCGCGCGCAGG
CGGCTTCTTAAGTCTGATGTGAAAGCCCACGGCTCAACCGTGGAGGGTCATTGGAAACTGGGAGGCTTGAGTGCAGAAGA
GGAGAGCGGAATTCCACGTGTAGCGGTGAAATGCGTAGAGATGTGGAGGAACACCAGTGGCGAAGGCGGCTCTCTGGTCT
GTAACTGACGCTGAGGCGCGAAAGCGTGGGGAGCGAACAGGATTAGATACCCTGGTAGTCCACGCCGTAAACGATGAGT
GCTAAGTGTTGGAGGGTTTCCGCCCTTCAGTGCTGCAGCTAACGCATTAAGCACTCCGCCTGGGGAGTACGGCCGCAAGGC
TGAAACTCAAAGGAATTGACGGGGGCCCGCACAAGCGGTGGAGCATGTGGTTTAATTCGAAGCAACGCGAAGAACCTTAC
CAGGTCTTGACATCCCGCTGACCGGTCTGGAGACAGGCCTTTCCCTTCGGGGACAGCGGTGACAGGTGGTGCATGGTTGTC
GTCAGCTCGTGTCGTGAGATGTTGGGTTAAGTCCCGCAACGAGCGCAACCCTTGATCTTAGTTGCCAGCATTCAGTTGGGC
ACTCTAAGGTGACTGCCGGTGACAAACCGGAGGAAGGTGGGGATGACGTCAAATCATCATGCCCCTTATGACCTGGGCTA
CACACGTGCTACAATGGATGGTACAAAGGGCTGCAAGACCGCAAGGTTTAGCCAATCCCATAAAACCATTCTCAGTTCGG
ATTGCAGGCTGCAACTCGCCTGCATGAAGCCGGAATCGCTAGTAATCGCGGATCAGCATGCCGCGGTGAATACGTTCCCG
GGCCTTGTACACACCGCCCGTCACACCACGACGAGTTTGCAACACCCGAAGTCGGTGGGGTCAACCCTGACGCGACCCAG
CCGACCTATAGAGGTACCCAGTG

N21:

GGACGTAGGGGGATGCCTACGCATGCAAGTCGAACGAGGCCCTTCGGGGTTTAGTGGCGCACGGGTGCGTAACGCGTGGG
AATCTGCCCTTTGGTTCGGAATAACAGCTGGAAACGGCTGCTAATACCGGATGATGACGAAAGTCCAAAGATTTATCGCC
AGAGGATGAGCCCGCGTTGGATTAGGTAGTTGGTGGGGTAAAGGCCTACCAAGCCGACGATCCATAGCTGGTCTGAGAGG

ATGATCAGCCACACTGGGACTGAGACACGGCCCAGACTCCTACGGGAGGCAGCAGTGGGGAATATTGGACAATGGGCGC
AAGCCTGATCCAGCAATGCCGCGTGAGTGATGAAGGCCCTAGGGTTGTAAAGCTCTTTTACCCGGGAAGATAATGACTGT
ACCGGGAGAATAAGCCCCGGCTAACTCCGTGCCAGCAGCCGCGGTAATACGGAGGGGGCTAGCGTTGTTCGGAATTACTG
GGCGTAAAGCGCACGTAGGCGGCTTTGTAAGTCAGAGGTGAAAGCCTGGAGCTCAACTCCAGAACTGCCTTTGAGACTGC
ATCGCTTGAATCCAGGAGAGGTCAGTGGAATTCCGAGTGTAGAGGTGAAATTCGTAGATATTCGGAAGAACACCAGTGGC
GAAGGCGGCTGACTGGACTGGTATTGACGCTGAGGTGCGAAAGCGTGGGGAGCAAACAGGATTAGATACCCTGGTAGTCC
ACGCCGTAAACGATGATAACTAGCTGTCCGGGCACTTGGTGCTTGGGTGGCGCAGCTAACGCATTAAGTTATCCGCCTGGG
GAGTACGGCCGCAAGGTTAAAACTCAAAGGAATTGACGGGGGCCTGCACAAGCGGTGGAGCATGTGGTTTAATTCGAAGC
AACGCGCAGAACCTTACCAGCGTTTGACATGTCCGGACGACTTCCAGAGATGGATTTCTTCCCTTCGGGGACTGGAACACA
GGTGCTGCATGGCTGTCGTCAGCTCGTGTCGTGAGATGTTGGGTTAAGTCCCGCAACGAGCGCAACCCTCGCCTTTAGTTA
CCATCATTTGGTTGGGTACTCTAAAGGAACCGCCGGTGATAAGCCGGAGGAAGGTGGGGATGACGTCAAGTCCTCATGGC
CCTTACGCGCTGGGCTACACACGTGCTACAATGGCAACTACAGTGGGCAGCGACCCTGCGAGGGCGAGCTAATCCCCAAA
AGTTGTCTCAGTTCGGATTGTTCTCTGCAACTCGAGAGCATGAAGGCGGAATCGCTAGTAATCGCGGATCAGCATGCCGCG
GTGAATACGTTCCCAGGCCTTGTACACACCGCCCGTCACACCATGGGAGTTGGATTCACCCGAAGGCGTTGCGCCAACCCG
TAAGGAAGCAGGCGACCACAGTGTACCCCG

N22:

GGGGCATGGCGGCAGCTACCATGCAGTCGAGCGGCAGCGGGAAGTAGCTTGCTACTTTGCCGGCGAGCGAGCGGACGGGT
GAGTAATGTCTGGGAAACTGCCTGATGGAGGGGGATAACTACTGGAAACGGTAGCTAATACCGCATAACGTCGCAAGACC
AAAGAGGGGGACCTTCGGGCCTCTTGCCATCAGATGTGCCCAGATGGGATTAGCTAGTAGGTGGGGTAATGGCTCACCTA
GGCGACGATCCCTAGCTGGTCTGAGAGGATGACCAGCCACACTGGAACTGAGACACGGTCCAGACTCCTACGGGAGGCAG
CAGTGGGGAATATTGCACAATGGGCGCAAGCCTGATGCAGCCATGCCGCGTGTATGAAGAAGGCCTTCGGGTTGTAAAGT
ACTTTCAGCGGGGAGGAAGGTGTTGAGGTTAATAACCTCAGCAATTGACGTTACCCGCAGAAGAAGCACCGGCTAACTCC
GTGCCAGCAGCCGCGGTAATACGGAGGGTGCAAGCGTTAATCGGAATTACTGGGCGTAAAGCGCACGCAGGCGGTCTGTC
AAGTCGGATGTGAAATCCCCGGGCTCAACCTGGGAACTGCATTCGAAACTGGCAGGCTAGAGTCTTGTAGAGGGGGGTAG
AATTCCAGGTGTAGCGGTGAAATGCGTAGAGATCTGGAGGAATACCGGTGGCGAAGGCGGCCCCCTGGACAAAGACTGA
CGCTCAGGTGCGAAAGCGTGGGGAGCAAACAGGATTAGATACCCTGGTAGTCCACGCCGTAAACGATGTCGACTTGGAGG
TTGTGCCCTTGAGGCGTGGCTTCCGGAGCTAACGCGTTAAGTCGACCGCCTGGGGAGTACGGCCGCAAGGTTAAAACTCA
AATGAATTGACGGGGGCCCGCACAAGCGGTGGAGCATGTGGTTTAATTCGATGCAACGCGAAGAACCTTACCTACTCTTG
ACATCCAGAGAACTTAGCAGAGATGCTTTGGTGCCTTCGGGAACTCTGAGACAGGTGCTGCATGGCTGTCGTCAGCTCGTG
TTGTGAAATGTTGGGTTAAGTCCCGCAACGAGCGCAACCCTTATCCTTTGTTGCCAGCGGTTCGGCCGGGAACTCAAAGGA
GACTGCCAGTGATAAACTGGAGGAAGGTGGGGATGACGTCAAGTCATCATGGCCCTTACGAGTAGGGCTACACACGTGCT
ACAATGGCGCATACAAAGAGAAGCGACCTCGCGAGAGCAAGCGGACCTCATAAAGTGCGTCGTAGTCCGGATTGGAGTCT
GCAACTCGACTCCATGAAGTCGGAATCGCTAGTAATCGTAGATCAGAATGCTACGGTGAATACGTTCCCGGGCCTTGTACA
CACCGCCCGTCACATCCATGGGCAGTGGGTTGCAAAAGAAGTAGGTAGCTTAACCTTCGGGAGGGCGCTACCTCTTGGAA
ATCCCCCC

N23:

GGGTTAGGGATACTATAGAGTGAGATCGTGCTCTCGATATCTCGTGGCGCTGGCTCCCTAGAGTGTTAGCGGCGGACGGG
AGAGTAACACGTAGGCAACCTGCCCGTAAGACTGGGATAACTACCGGAAACGGTAGCTAATACCGGATACGCAAGTTTCT
CGCATGAGGGGCTTGGGAAAGGCGGAGCAATCTGTCACTTACGGATGGGCCTGCGGCGCATTAGCTAGTAGGTGGGGTAA
CGGCCTACCTAGGCGACGATGCGTAGCCGACCTGAGAGGGTGAACGGCCACACTGGGACTGAGACACGGCCCAGACTCCT
ACGGGAGGCAGCAGTAGGGAATCTTCCGCAATGGACGAAAGTCTGACGGAGCAACGCCGCGTGAGTGATGAAGGTTTTCG
GATCGTAAAGCTCTGTTGCCAGGGAAGAACGTCCTATAGAGTAACTGCTCTCAGAGTGACGGTACCTGAGAAGAAAGCCC
CGGCTAACTACGTGCCAGCAGCCGCGGTAATACGTAGGGGGCGAGCGTTGTCCGGAATTATTGGGCGTAAAGCGCGCGCA

GGCGGCTGTTTAAGTCTGGTGTTTAATCCTGGGGCTCAACCCCGGGTCGCACTGGAAACTGGACAGCTTGAGTGCAGAAG
AGGAGAGTGGAATTCCACGTGTAGCGGTGAAATGCGTAGAGATGTGGAGGAACACCAGTGGCGAAGGCGACTCTCTGGG
CTGTAACTGACGCTGAGGCGCGAAAGCGTGGGGAGCAAACAGGATTAGATACCCTGGTAGTCCACGCCGTAAACGATGAA
TGCTAGGTGTTAGGGGTTTCGATACCCTTGGTGCCGAAGTTAACACATTAAGCATTCCGCCTGGGGAGTACGGCCGCAAGG
CTGAAACTCAAAGGAATTGACGGGGACCCGCACAAGCAGTGGAGTATGTGGTTTAATTCGAAGCAACGCGAAGAACCTTA
CCAGGTCTTGACATCCCCCTGACCGGTTCAGAGATGTTCCTTTCCTTCGGGACAGGGGAGACAGGTGGTGCATGGTTGTCG
TCAGCTCGTGTCGTGAGATGTTGGGTTAAGTCCCGCAACGAGCGCAACCCTTGACTTTAGTTGCCAGCAGGTCAGGCTGGG
CACTCTAGAGTGACTGCCGGTGACAAACCGGAGGAAGGTGGGGATGACGTCAAATCATCATGCCCCTTATGACCTGGGCT
ACACACGTACTACAATGGCCGGTACAACGGGAAGCGAAGGAGCGATCTGGAGCGAATCCTTGAAAGCCGGTCTCAGTTCG
GATTGCAGGCTGCAACTCGCCTGCATGAAGTCGGAATTGCTAGTAATCGCGGATCAGCATGCCGCGGTGAATACGTTCCCG
GGTCTTGTACACACCGCCCGTCACAGCAACAGGGTATCCAACGAGTGAAGCCGGTCTCTATATGTGTTGACACAATATCCC
TAGAGATGTATGCC

P212:

TCCGTAGGTGAACCTGCGGAAGGATCATTACAGAATTTTGAGAATTGTGCTTAACTGCACTTTTCTTATCTACACACGTGTT
TTTGTTTTATTCTTAAAACTTGCTTTGGCAGTGGCTGCTTAATTGCTCTGCTGCCAGAGGATAAACTCAACCTAAATTTTTA
TTTTAAACTAGTCAACTGATTATATTTATTAATAGTCAAAACTTTCAACAACGGATCTCTTGGTTCTCGCATCGATGAAGAA
CGCAGCGAAATGCGATAAGTAATATGAATTGCAGATATTCGTGAATCATCGAATCTTTGAACGCACATTGCGCCCTCTGGT
ATTCCGGAGGGCATGCCTGTTTGAGCGTCATTTCTCCCTCAAACCCCCGGGTTTGGTGATGAGCAATACGCCAGGTTTGCTT
GAAAGTTAGGAGGAGTATTTATAACAATGTATTAGGTCTAACCACTCCATTGTGCTTAATAAAAGCTCCAATCTATATTTC
AAACTTCGACCTCAAATCAGGTAGGATTACCCGCTGAACTTAAGCATATCAATAAGCGGAGGAA

N224:

GGGCAAGGGTATACTATAGAGTGAGATCGTGCTCTCGATGTGTCGTAACAGGGTCCCCCTAGAGTGTGATCGAGGGGCGA
GTAACACGTGGGTAACCTGCCTGTAAAACTGGGATAACTCCGGGAAACCGGAGCTAATACCGGATAGTTCCTTGAACCGC
ATGGTTCAAGGATGAAAGACGGTTTCGGCTGTCACTTACAGATGGACCCGCGGCGCATTAGCTAGTTGGTGAGGTAACGG
CTCACCAAGGCGACGATGCGTAGCCGACCTGAGAGGGTGATCGGCCACACTGGGACTGAGACACGGCCCAGACTCCTACG
GGAGGCAGCAGTAGGGAATCTTCCGCAATGGACGAAAGTCTGACGGAGCAACGCCGCGTGAGTGATGAAGGTTTTCGGAT
CGTAAAGCTCTGTTGTTAGGGAAGAACAAGTGCAAGAGTAACTGCTTGCACCTTGACGGTACCTAACCAGAAAGCCACGG
CTAACTACGTGCCAGCAGCCGCGGTAATACGTAGGTGGCAAGCGTTGTCCGGAATTATTGGGCGTAAAGGGCTCGCAGGC
GGTTTCTTAAGTCTGATGTGAAAGCCCCCGGCTCAACCGGGGAGGGTCATTGGAAACTGGGAAACTTGAGTGCAGAAGAG
GAGAGTGGAATTCCACGTGTAGCGGTGAAATGCGTAGAGATGTGGAGGAACACCAGTGGCGAAGGCGACTCTCTGGTCTG
TAACTGACGCTGAGGAGCGAAAGCGTGGGGAGCGAACAGGATTAGATACCCTGGTAGTCCACGCCGTAAACGATGAGTG
CTAAGTGTTAGGGGGTTTCCGCCCCTTAGTGCTGCAGCTAACGCATTAAGCACTCCGCCTGGGGAGTACGGTCGCAAGACT
GAAACTCAAAGGAATTGACGGGGGCCCGCACAAGCGGTGGAGCATGTGGTTTAATTCGAAGCAACGCGAAGAACCTTACC
AGGTCTTGACATCCTCTGACAACCCTAGAGATAGGGCTTTCCCTTCGGGGACAGAGTGACAGGTGGTGCATGGTTGTCGTC
AGCTCGTGTCGTGAGATGTTGGGTTAAGTCCCGCAACGAGCGCAACCCTTGATCTTAGTTGCCAGCATTCAGTTGGGCACT
CTAAGGTGACTGCCGGTGACAAACCGGAGGAAGGTGGGGATGACGTCAAATCATCATGCCCCTTATGACCTGGGCTACAC
ACGTGCTACAATGGACAGAACAAAGGGCTGCGAGACCGCAAGGTTTAGCCAATCCCACAAATCTGTTCTCAGTTCGGATC
GCAGTCTGCAACTCGACTGCGTGAAGCTGGAATCGCTAGTAATCGCGGATCAGCATGCCGCGGTGAATACGTTCCCGGGC
GTTGTACACACCGCCCGTCACTCCAACACGGTAACCCCAGAGTTTGATCGCGGCTCTCTATATGTCGACACAGGTATCACC
AGAGAGTACTGCG

P292:

TTCCTCCGCTTATTGATATGCTTAAGTTCAGCGGGTAATCCTACCTGATTTGAGGTCGAAGTTTGAAATATAGATTGGAGCT
TTTATTAAGCACAATGGAGTGGTTAGACCTAATACATTGTTATAAATACTCCTCCTAACTTTCAAGCAAACCTGGCGTATTG

CTCATCACCAAACCCGGGGGTTTGAGGGAGAAATGACGCTCAAACAGGCATGCCCTCCGGAATACCAGAGGGCGCAATGT
GCGTTCAAAGATTCGATGATTCACGAATATCTGCAATTCATATTACTTATCGCATTTCGCTGCGTTCTTCATCGATGCGAGA
ACCAAGAGATCCGTTGTTGAAAGTTTTGACTATTAATAAATATAATCAGTTGACTAGTTTAAAATAAAAAATTTAGGTTGA
GTTTATCCTCTGGCAGCAGAGCAATTAAGCAGCCACTGCCAAAGCAAGTTTTAAGAATAAAACAAAAACACGTGTGTAGA
TAAGAAAAGTGCAGTTAAGCACAATTCTCAAAATTCTGTAATGATCCTTCCGCAGGTTCACCTACGGAAG

D215:

TTTCCTCCGGGCCTTTGATATGCTTAAGTTCAGCGGGTATTCCTACCTGATCCGAGGTCAACCTGTAAAGAATTTGGGGGTT
TAACGGCAAGAGTCCCTCCGGATCCCAGTGCGAGACGTTAGTTACTACGCAAAGGAGGCTCCGGGAGGGTCCGCCACTAC
CTTTAAGGGCCCACGTCGGCCGTGGGGCCCCAAAACCAAGCGGTGCTTGAGGGTTGAAATGACGCTCGAACAGGCATGCT
CGCCAGAATGCTGGCGAGCGCAATGTGCGTTCAAAGATTCGATGATTCACTGAATTCTGCAATTCACATTACTTATCGCAT
TTCGCTGCGTTCTTCATCGATGCCAGAACCAAGAGATCCGTTGTTAAAAGTTTTAATTATTTGCTTGTGCCACTCAGAAGAG
ACGTCGTGTAAATAGAGTTTGGTTTCCTCCGGCGGGCGCCCCGTCCCCGTGGTGGGGGCCGGCGCCGGGAGGGGAGGCCC
GCGAGAGGCTTCCCCTGCCCGCCGAAGCAACGGTTAGGTACGTTCACAAAGGGTTATAGAGCGGTAACTCAGTAATGATC
CCTCCGCAGGTACCCCTTACGGAA

P2102:

TTCCTCCGCTTATTGATATGCTTAAGTTCAGCGGGTAATCCTACCTGATTTGAGGTCGAAGTTTGAAATATAGATTGGAGCT
TTTATTAAGCACAATGGAGTGGTTAGACCTAATACATTGTTATAAATACTCCTCCTAACTTTCAAGCAAACCTGGCGTATTG
CTCATCACCAAACCCGGGGGTTTGAGGGAGAAATGACGCTCAAACAGGCATGCCCTCCGGAATACCAGAGGGCGCAATGT
GCGTTCAAAGATTCGATGATTCACGAATATCTGCAATTCATATTACTTATCGCATTTCGCTGCGTTCTTCATCGATGCGAGA
ACCAAGAGATCCGTTGTTGAAAGTTTTGACTATTAATAAATATAATCAGTTGACTAGTTTAAAATAAAAAATTTAGGTTGA
GTTTATCCTCTGGCAGCAGAGCAATTAAGCAGCCACTGCCAAAGCAAGTTTTAAGAATAAAACAAAAACACGTGTGTAGA
TAAGAAAAGTGCAGTTAAGCACAATTCTCAAAATTCTGTAATGATCCTTCCGCAGGTTCACCTACGGAAG

S216:

TTCCGTAGGTGAACCTGCGGAGGGATCATTACTGAGTTACCGCTCTATAACCCTTTGTGAACGTACCTAACCGTTGCTTCG
GCGGGCAGGGGAAGCCTCTCGCGGGCCTCCCCTCCCGGCGCCGGCCCCCACCACGGGGACGGGGCGCCCGCCGGAGGAA
ACCAAACTCTATTTACACGACGTCTCTTCTGAGTGGCACAAGCAAATAATTAAAACTTTTAACAACGGATCTCTTGGTTCT
GGCATCGATGAAGAACGCAGCGAAATGCGATAAGTAATGTGAATTGCAGAATTCAGTGAATCATCGAATCTTTGAACGCA
CATTGCGCTCGCCAGCATTCTGGCGAGCATGCCTGTTCGAGCGTCATTTCAACCCTCAAGCACCGCTTGGTTTTGGGGCCCC
ACGGCCGACGTGGGCCCTTAAAGGTAGTGGCGGACCCTCCCGGAGCCTCCTTTGCGTAGTAACTAACGTCTCGCACTGGGA
TCCGGAGGGACTCTTGCCGTTAAACCCCCAAATTCTTTACAGGTTGACCTCGGATCAGGTAGGAATACCCGCTGAACTTAA
GCATATCAAAAGCCGGGAGGA

261:

GGCCGAGGCGACACCATAGCGTGCGAGTCGCTCGATGAATCCCTTCGGGGTGGATTAGTGGCGAACGGGTGAGTAACACG
TGGGCAATCTGCCCTTCACTCTGGGACAAGCCCTGGAAACGGGGTCTAATACCGGATACGACCTTCGAGCGCATGCTTGAA
GGTGGAAAGCTCCGGCGGTGAAGGATGAGCCCGCGGCCTATCAGCTTGTTGGTGGGGTGATGGCCTACCAAGGCGACGAC
GGGTAGCCGGCCTGAGAGGGCGACCGGCCACACTGGGACTGAGACACGGCCCAGACTCCTACGGGAGGCAGCAGTGGGG
AATATTGCACAATGGGCGAAAGCCTGATGCAGCGACGCCGCGTGAGGGATGACGGCCTTCGGGTTGTAAACCTCTTTCAG
CAGGGAAGAAGCGAAAGTGACGGTACCTGCAGAAGAAGCGCCGGCTAACTACGTGCCAGCAGCCGCGGTAATACGTAGG
GCGCAAGCGTTGTCCGGAATTATTGGGCGTAAAGAGCTCGTAGGCGGCTTGTCGCGTCGGATGTGAAAGCCCGGGGCTTA
ACCCCGGGTCTGCATTCGATACGGGCAGGCTAGAGTTCGGTAGGGGAGATCGGAATTCCTGGTGTAGCGGTGAAATGCGC
AGATATCAGGAGGAACACCGGTGGCGAAGGCGGATCTCTGGGCCGATACTGACGCTGAGGAGCGAAAGCGTGGGGAGCG
AACAGGATTAGATACCCTGGTAGTCCACGCCGTAAACGTTGGGAACTAGGTGTGGGCGACATTCCACGTCGTCCGTGCCG

CAGCTAACGCATTAAGTTCCCCGCCTGGGGAGTACGGCCGCAAGGCTAAAACTCAAAGGAATTGACGGGGGCCCGCACAA
GCAGCGGAGCATGTGGCTTAATTCGACGCAACGCGAAGAACCTTACCAAGGCTTGACATACACCGGAAACGGCCAGAGAT
GGTCGCCCCCTTGTGGTCGGTGTACAGGTGGTGCATGGCTGTCGTCAGCTCGTGTCGTGAGATGTTGGGTTAAGTCCCGCA
ACGAGCGCAACCCTTGTCCTGTGTTGCCAGCATGCCCTTCGGGGTGATGGGGACTCACAGGAGACTGCCGGGGTCAACTC
GGAGGAAGGTGGGGACGACGTCAAGTCATCATGCCCCTTATGTCTTGGGCTGCACACGTGCTACAATGGCCGGTACAATG
AGCTGCGATACCGTGAGGTGGAGCGAATCTCAAAAAGCCGGTCTCAGTTCGGATTGGGGTCTGCAACTCGACCCCATGAA
GTTGGAGTTGCTAGTAATCGCAGATCAGCATTGCTGCGGTGAATACGTTCCCGGGCCTTGTACACACCGCCCGTCACGTCA
CGAAAGTCGGTAACACCGGAAGCCGGTGGCTCCAACCATGGTGGACACAGCGTCACGCTATAGAGTGCCCCCCCT

262:

GCAAGGGGCAGCATAGCATGCAGTCGAGCGGTAGCACAGGGGAGCTTGCTCCCTGGGTGACGAGCGGCGGACGGGTGAG
TAATGTCTGGGAAACTGCCTGATGGAGGGGGATAACTACTGGAAACGGTAGCTAATACCGCATAACGTCGCAAGACCAAA
GAGGGGGACCTTCGGGCCTCTTGCCATCAGATGTGCCCAGATGGGATTAGCTAGTAGGTGGGGTAATGGCTCACCTAGGC
GACGATCCCTAGCTGGTCTGAGAGGATGACCAGCCACACTGGAACTGAGACACGGTCCAGACTCCTACGGGAGGCAGCAG
TGGGGAATATTGCACAATGGGCGCAAGCCTGATGCAGCCATGCCGCGTGTGTGAAGAAGGCCTTCGGGTTGTAAAGCACT
TTCAGCGAGGAGGAAGGTGGTGAGCTTAATACGCTCATCAATTGACGTTACTCGCAGAAGAAGCACCGGCTAACTCCGTG
CCAGCAGCCGCGGTAATACGGAGGGTGCAAGCGTTAATCGGAATTACTGGGCGTAAAGCGCACGCAGGCGGTTTGTTAAG
TCAGATGTGAAATCCCCGGGCTCAACCTGGGAACTGCATTTGAAACTGGCAAGCTAGAGTCTCGTAGAGGGGGGTAGAAT
TCCAGGTGTAGCGGTGAAATGCGTAGAGATCTGGAGGAATACCGGTGGCGAAGGCGGCCCCCTGGACGAAGACTGACGCT
CAGGTGCGAAAGCGTGGGGAGCAAACAGGATTAGATACCCTGGTAGTCCACGCTGTAAACGATGTCGATTTGGAGGTTGT
GCCCTTGAGGCGTGGCTTCCGGAGCTAACGCGTTAAATCGACCGCCTGGGGAGTACGGCCGCAAGGTTAAAACTCAAATG
AATTGACGGGGGCCCGCACAAGCGGTGGAGCATGTGGTTTAATTCGATGCAACGCGAAGAACCTTACCTACTCTTGACAT
CCAGAGAACTTTCCAGAGATGGATTGGTGCCTTCGGGAACTCTGAGACAGGTGCTGCATGGCTGTCGTCAGCTCGTGTTGT
GAAATGTTGGGTTAAGTCCCGCAACGAGCGCAACCCTTATCCTTTGTTGCCAGCGGTTCGGCCGGGAACTCAAAGGAGACT
GCCAGTGATAAACTGGAGGAAGGTGGGGATGACGTCAAGTCATCATGGCCCTTACGAGTAGGGCTACACACGTGCTACAA
TGGCGTATACAAAGAGAAGCGACCTCGCGAGAGCAAGCGGACCTCATAAAGTACGTCGTAGTCCGGATTGGAGTCTGCAA
CTCGACTCCATGAAGTCGGAATCGCTAGTAATCGTAGATCAGAATGCTACGGTGAATACGTTCCCGGGCCTTGTACACACC
GCCCGTCACACCATGGGAGTGGGTTGCAAAAGAAGTAGGTAGCTTAACCTTCGGGAGGGCGCTACCACTTTGATCCAGCC
CG
